# Crucial Contributions by T Lymphocytes (Effector, Regulatory, and Checkpoint Inhibitor) and Cytokines (TH1, TH2, and TH17) to a Pathological Complete Response Induced by Neoadjuvant Chemotherapy in Women with Breast Cancer

**DOI:** 10.1155/2016/4757405

**Published:** 2016-09-29

**Authors:** Viriya Kaewkangsadan, Chandan Verma, Jennifer M. Eremin, Gerard Cowley, Mohammed Ilyas, Oleg Eremin

**Affiliations:** ^1^Division of Gastrointestinal Surgery, Nottingham Digestive Diseases Centre, Faculty of Medicine and Health Sciences, University of Nottingham, E Floor, West Block, Queen's Medical Centre, Derby Rd, Nottingham NG7 2UH, UK; ^2^Research & Development Department, Lincoln Breast Unit, Lincoln County Hospital, Greetwell Road, Lincoln LN2 5QY, UK; ^3^Department of Pathology, PathLinks, Lincoln County Hospital, Greetwell Road, Lincoln LN2 5QY, UK; ^4^Academic Department of Pathology, Faculty of Medicine and Health Sciences, University of Nottingham, A Floor, West Block, Queens Medical Centre, Derby Road, Nottingham NG7 2UH, UK

## Abstract

The tumour microenvironment consists of malignant cells, stroma, and immune cells. Prominent tumour-infiltrating lymphocytes (TILs) in breast cancer are associated with a good prognosis and are predictors of a pathological complete response (pCR) with neoadjuvant chemotherapy (NAC). The contribution of different T effector/regulatory cells and cytokines to tumour cell death with NAC requires further characterisation and was investigated in this study. Breast tumours from 33 women with large and locally advanced breast cancers undergoing NAC were immunohistochemically (intratumoural, stromal) assessed for T cell subsets and cytokine expression using labelled antibodies, employing established semiquantitative methods. Prominent levels of TILs and CD4^+^, CD8^+^, and CTLA-4^+^ (stromal) T cells and CD8^+^ : FOXP3^+^ ratios were associated with a significant pCR; no association was seen with FOXP3^+^, CTLA-4^+^ (intratumoural), and PD-1^+^ T cells. NAC significantly reduced CD4^+^, FOXP3^+^, CTLA-4^+^ (stromal) (concurrently blood FOXP3^+^, CTLA-4^+^ Tregs), and PD-1^+^ T cells; no reduction was seen with CD8^+^ and CTLA-4^+^ (intratumoural) T cells. High post-NAC tumour levels of FOXP3^+^ T cells, IL-10, and IL-17 were associated with a failed pCR. Our study has characterised further the contribution of T effector/regulatory cells and cytokines to tumour cell death with NAC.

## 1. Background

The induction, development, and dissemination of malignant disease in man are complex processes involving a crucial interplay between malignant cells, surrounding stroma and tumour-infiltrating inflammatory and immune cells [1–3]. In a range of human solid tumours, variable numbers of innate and adaptive immune cells have been documented in the tumour microenvironment (tumour cell nests, peritumoural stroma). The distribution and density of the immune cells vary between different histopathological cancer types and amongst cancers of the same type. In general, however, they are present at increased levels compared with nonmalignant tissues [2, 4, 5].

A number of studies have shown that the presence of a prominent lymphocytic infiltrate in tumours is associated with an improved prognosis and good long-term clinical outcome in patients with different types of cancer [2, 4–7]. The presence of tumour-infiltrating lymphocytes (TILs) has been recognised as a biomarker of an antitumour response in a wide range of solid cancers (breast, bowel, renal, and melanoma) [2, 8]. In breast cancer it has been shown that a prominent TIL presence is associated with an increased incidence of a pathological complete response (pCR) in the tumour following neoadjuvant chemotherapy (NAC) [9–11]. The subsets of T cells (CD4^+^, CD8^+^, FOXP3^+^(forkhead box protein 3), and PD-1^+^(programmed death molecule 1)) infiltrating breast cancer, however, can have different pathobiological significance and prognostic characteristics and are a matter of continuing debate [2, 5, 12–16]. The interrelationship between NAC and the various subsets is a matter of great scientific and clinical interest. It is, however, not well characterised and is in need of further study to define more precisely its contribution to a possible immune-mediated tumour cell death with NAC [17–20].

We have previously reported that women with large and locally advanced breast cancers (LLABCs) have a significantly increased circulating level of T regulatory cells (Tregs). The % of FOXP3^+^ Tregs correlated with the pathological response of the LLABCs to subsequent NAC. Following NAC the blood Tregs (%) were significantly reduced in women whose tumours showed a good pathological response. We also documented polarised T helper cell (Th1, Th2, and Th17) profiles in the blood lymphocytes but these were unaltered by NAC [21]. There is evidence that the host anticancer immune response, at both the molecular and cellular levels, varies in different anatomical compartments and that the molecular and cellular changes detected in the blood may not always reflect the situation in the tumour microenvironment [22].

We wished, therefore, to investigate the tumour microenvironment in LLABCs and to establish whether there was a concomitant anticancer immune response, and if the blood immune changes associated with NAC were reflected in comparable changes in the tumour microenvironment. We carried out an immunohistochemical analysis of various lymphocytic immune cells and humoral factors infiltrating LLABCs. We documented the pathological impact of NAC on the tumour microenvironment and the possible contribution of different host immune cells and humoral factors to an immune-mediated tumour cell death and pCR to NAC, a recognised surrogate marker of a beneficial long-term clinical response in breast cancer [23, 24].

## 2. Materials and Methods

### 2.1. Patients and Samples

Paraffin-embedded sections of breast tumours from 33 women with L (≥3 cm), LABCs (T3, 4; N1, 2; M0), enrolled in a study of NAC (between 2008 and 2011) were studied [24].

Histological diagnosis was established from ultrasound-guided core biopsies. To minimise tumour heterogeneity and sampling discrepancies, several core biopsies were obtained from each tumour. All tumours prior to NAC had a radioopaque coil inserted. After NAC, wire-guided removal of the residual “tumour” was carried out (in the case of breast conservation) if there was no clinical or radiological evidence of cancer. Operative specimens (wide local excision, mastectomy) had radiological confirmation of the presence of the coil to ensure accurate localisation and histopathological evaluation. Representative tissue sections were used for immunohistochemical evaluation. All pre- and post-NAC specimens were discussed at a multidisciplinary meeting and a consensus was reached about the pathological response and treatment options.

The NAC trial evaluated the effect of the addition of capecitabine (X) to docetaxel (T) preceded by adriamycin and cyclophosphamide (AC). All patients received either 4 courses of AC followed by 4 courses of T ± X or 2 courses of AC followed by 6 courses of T ± X, as per the trial protocol. Pathological responses were assessed in the excised surgical specimens after NAC. Established and previously published grading criteria were used to define histopathological responses in the breast [25, 26]. Good pathological responses were graded 5 (pCR, no residual invasive disease) and 4 (90% loss of invasive disease). Poor pathological responses were graded as 3 (30–90% loss of invasive disease), 2 (<30% loss of invasive disease), and 1 (no loss of tumour cells). The levels of blood FOXP3^+^ and CTLA-4^+^ Tregs from 16 of these 33 patients have been documented in a previous study from our department [21]. An important aim of the work presented was to establish whether the previously documented inhibition of the blood Tregs by NAC occurred concurrently in the breast tumours of the same individuals. Patient cases were randomly selected based on availability of tissue specimens and equability of distribution between compared groups (pCR versus non-pCR).

### 2.2. Immunohistochemical Assessment

Immunohistochemical (IHC) assessments of immune cell subsets and expression of cytokines were performed in 4 *μ*m tissue sections. Briefly, paraffin-embedded tissue sections were dewaxed and rehydrated using xylene and graded alcohol. Citrate buffer, pH 6.0, at 98°C was added for 20 minutes (mins) for antigen retrieval. After serial blocking, the sections were incubated with the primary monoclonal antibody (MAb) against CD4 (Dako, M7310, clone 4B12), 1 : 80 dilution for 30 mins at room temperature (RT); MAb against CD8 (Dako, M7103, clone C8/144B), 1 : 100 dilution for 30 mins at RT; MAb against FOXP3 (Abcam, ab20034, clone 236A/E7), 20 *μ*g/mL for 30 mins at RT; MAb against CTLA-4 (Santa Cruz Bio, sc-376016, clone F-8), 1 : 300 dilution for 30 mins at RT; MAb against PD-1 (Abcam, ab52587, clone NAT105), 1 : 100 dilution for 30 mins at RT; MAb against interleukin-1 (IL-1) (Abcam, ab8320, clone 11E5), 1 : 150 dilution overnight at 4°C; MAb against IL-2 (Abcam, ab92381, clone EPR2780), 1 : 500 dilution for 30 mins at RT; polyclonal Ab against IL-4 (Abcam, ab9622), 4 *μ*g/mL for 30 mins at RT; polyclonal Ab against IL-10 (Abcam, ab34843), 1 : 400 dilution for 30 mins at RT; polyclonal Ab against IL-17 (Abcam, ab9565), 1 : 100 dilution for 30 mins at RT; polyclonal Ab against interferon-gamma (IFN-*γ*) (Abcam, ab9657), 4 *μ*g/mL for 30 mins at RT; MAb against transforming growth factor-beta 1 (TGF-*β*1) (Abcam, ab64715, clone 2Ar2), 12 *μ*g/mL overnight at 4°C; polyclonal Ab against PD-L1 (Abcam, ab58810), 2.5 *μ*g/mL for 15 mins at RT. The Novolink*™* polymer detection system, Leica RE7280-K with polymeric horseradish peroxidase- (HRP-) linker antibody conjugates and diaminobenzidine (DAB) chromogen, was used for enzyme-substrate labelling. Finally, the sections were counterstained with haematoxylin, dehydrated, and mounted in DPX mounting medium. Positive and negative staining controls were carried out with tonsil sections except for CTLA-4 (colon carcinoma sections), IL-1, IL-4, and TGF-*β* (kidney carcinoma sections), and IL-10 (normal colon sections). Negative staining controls were demonstrated by omitting the primary antibody. Positive and negative controls were simultaneously performed with every IHC staining run.

### 2.3. Semiquantification of IHC Sections

Whole tissue sections were studied rather than microarrays (to minimise sampling bias). Representative high-power fields (HPFs) ×400 magnification are shown for ease and clarity of presentation. To evaluate the presence and extent of specific T cell subsets in the breast tumours, the average numbers of brown membrane/nuclear-stained cells regardless of the intensity were counted in 5 HPFs. Positively stained cells in contact with tumour cells or within the tumour cell nests were defined as “intratumoural” whereas positively stained cells in the interstitial stroma surrounding tumour nests were defined as “stromal.” Evaluation of subset infiltrations in post-NAC specimens was undertaken on residual tumour nests and in the case of pCR (complete disappearance of invasive tumour cells in the specimen) in the tumour bed. The latter was characterised histologically as a hyalinised, amorphous area with haemosiderin deposits [27, 28].

To evaluate the presence of IL-1, IL-2, IL-4, IL-10, IL-17, IFN-*γ*, TGF-*β*, and PD-L1 the semiquantitative *H* scoring system was employed using whole tissue sections. The *H* score was calculated by multiplying the % of positive cells (tumour and immune) by a factor representing the intensity of immune-reactivity (1 for weak, 2 for moderate, and 3 for strong), giving a maximum score of 300. A score of <50 was considered negative and a score of 50–100 was considered weakly positive (1+). A score of 101–200 was regarded as moderately positive (2+) and a score of 201–300 as strongly positive (3+). Negative and 1+ were considered as low expression whereas 2+ and 3+ were considered as high expression.

To evaluate TILs in haematoxylin and eosin (H&E) stained sections, intratumoural lymphocytes (Itu-Ly) were reported as the % of the tumour epithelial nests that contained infiltrating lymphocytes. Stromal lymphocytes (Str-Ly) were defined as the % of tumour stromal areas that contained lymphocytic infiltrates without direct contact with tumour cells. Scores of >60% were considered to be high levels of infiltration, whilst ≤60% were considered to be low levels of infiltration for both Itu-Ly and Str-Ly. Cases were defined as high TILs when Itu-Ly and/or Str-Ly were >60% and as low TILs if both Itu-Ly and Str-Ly were ≤60%. The 60% cut-off point for level of TILs was following previously published studies and the methodological recommendations from the international TILs working group 2014 [9, 29, 30]. All sections were scored without knowledge of the patients' clinical and pathological parameters.

### 2.4. Phenotypic Analysis of Blood FOXP3^+^ and CTLA-4^+^ Tregs

Blood samples were collected before and following completion of NAC. Blood mononuclear cells (BMCs) were collected on Ficoll-Hypaque, washed and made up in RPMI with 10% foetal calf serum (FCS) (Sigma, UK) and antibiotics, and stored at −80°C for further analysis. Whole blood assays were used for documentation of absolute numbers (AbNs). Flow cytometry analysis (Beckman Coulter, FC500) was performed with a panel of MAbs. FOXP3^+^ Tregs were stained for cell surface markers for 30 mins with 2.5 *μ*L phycoerythrin Texas red conjugate- (ECD-) anti-human CD4, 5 *μ*L phycoerythrin-anti-human CD25, 5 *μ*L allophycocyanin-anti-human CD127; CTLA-4^+^ Tregs were stained for intracellular CD152. Cell surface markers for CD4 and CD25 were determined by staining for 30 mins with 2.5 *μ*L of ECD anti-human CD4 and 5 *μ*L fluorescein isothiocyanate (FITC) anti-human CD25. The cells were then washed with RPMI and 2% FCS; 2% formaldehyde was used for fixation of BMCs for 10 mins at RT. The BMCs were then washed once in phosphate buffered saline (PBS) containing 2% FCS and twice in PBS/0.5% Tween with 0.05% azide and 3% FCS. 2.5 *μ*L FITC anti-human FOXP3 (intracellular) and 5 *μ*L PE anti-human CTLA-4 (intracellular CD152) were added to the corresponding tubes and incubated for 2 hours at 4°C. The BMC pellet was then washed twice in PBS/0.5% Tween, 0.05% azide, and 3% FCS. The BMCs were resuspended in 400 *μ*L of 0.5% paraformaldehyde fixative solution for FC analysis. Whole blood was used to determine absolute numbers of cells. CD4^+^ CD25^+^ Tregs were characterised using 2.5 *μ*L ECD anti-human CD4 and 5 *μ*L of PE anti-human CD25. CTLA-4^+^ Tregs were characterised using 2.5 *μ*L ECD anti-human CD4, 5 *μ*L of FITC anti-human CD25, and 5 *μ*L PE anti-human CD152 (intracellular CTLA-4). On adding the MAbs to whole blood a gentle vortex was applied for 5 seconds and the FACs tubes were left in the dark for 15 mins at RT. 500 *μ*L of optilyse C solution (Beckman Coulter) was added to induce complete lysis of red blood cells, vortexed, and left for another 15 mins at RT in the dark. 500 *μ*L of PBS was added to the FACS tubes to stop the lysis reaction between the optilyse C and the whole blood. The whole blood mixture was vortexed at RT. 100 *μ*L of Flow Count-fluorosphere beads (Beckman Coulter) was added prior to analysis on the flow-cytometer.

### 2.5. Statistical Analysis

Statistical analyses were performed with the IBM SPSS statistics software, version 21 (SPSS Inc., Chicago, IL, USA). Where the data did not follow a normal distribution, nonparametric tests (Mann–Whitney *U* test (between two variables/groups) and Kruskal-Wallis test (amongst three or more variables/groups)) were used to compare the groups based on pathological responses and clinicopathological parameters. Pearson Chi-Square test was performed to compare the binomial data (negative/low versus high) on expression of cytokines between groups. To evaluate and compare the related-sample data between pre-NAC and post-NAC groups, the Related-Samples Wilcoxon Signed Rank test and Related-Samples McNemar test were performed for comparing the number of cell counts and the expression of cytokines/PD-L1, respectively. The correlations between TILs and T cell subsets (continuous data) and grade (1–5) of pathological responses (ordinal data) were carried out using Spearman's Correlation Coefficient (rho). A univariate and multivariate (logistic regression) analysis was carried out to establish predictive factors for a pCR with NAC. A probability value (*p* value) of equal to or less than 0.05 (2-tailed) was considered statistically significant. Based on our previous study with Treg findings and using the *N* Query Advisor 6.0 analysis software, we established that the minimum number of patients (*n* = 7) in a sample group relating to the pathological response groups was appropriate [21].

## 3. Results

### 3.1. Prominent Lymphocytic Infiltration (Intratumoural, Stromal) of LLABCs: Association with a Significant Pathological Complete Response (PCR) in the Tumour following NAC

**Figure 1:**
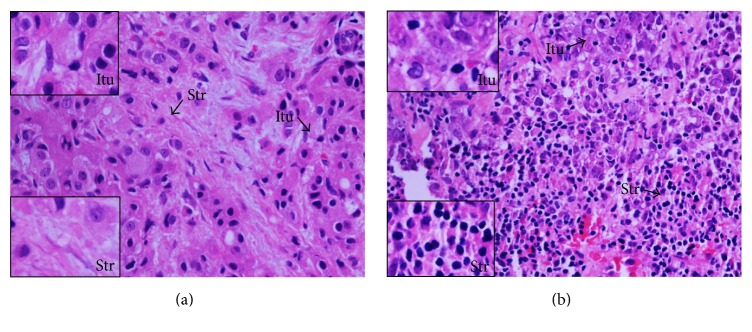
TILs in the sections of LLABCs, using H&E staining, at 400x magnification; (a) low level of lymphocytic infiltration; (b) high level of lymphocytic infiltration. Low level of TILs defined as ≤60% of tumour nests (Itu: intratumoural) and stromal areas (Str: stromal) infiltrated by lymphocytes. High level of TILs defined as >60% of tumour nests and/or stromal areas infiltrated by lymphocytes.

High levels of TILs were associated with a significant pCR (grade 5 response: no residual invasive disease in the breast cancer) following 8 cycles of NAC. This was seen with both intratumoural (*p* = 0.001) and stromal (*p* < 0.001) TILs and in lymphocyte predominant breast cancers (LPBCs) (*p* < 0.001), irrespective of the tumour microenvironment (Table 1) (Figure 1).

There was a significant positive correlation between pre-NAC intratumoural and stromal TILs (rho = 0.592, *p* = 0.016). There was also a significant positive correlation between post-NAC intratumoural and stromal TILs (rho = 0.693, *p* = 0.004). No significant difference, however, was found between levels of pre-NAC and post-NAC TILs (see Additional File 1 in Supplementary Material available online at http://dx.doi.org/10.1155/2016/4757405).

### 3.2. Prominent Intratumoural and Stromal CD4^+^and CD8^+^T Cell and Stromal CTLA-4^+^ T Cell Infiltration in LLABCs: Association with a Significant Pathological Complete Response (PCR) in the Tumour following NAC

Table 2 shows that high levels of intratumoural (tumour cell nests) infiltration by CD4^+^and CD8^+^T cells were associated with a significant pCR in the breast cancer (*p* = 0.023 and *p* = 0.008, resp.). Infiltration by FOXP3^+^, CTLA-4^+^, and PD-1^+^ T cells (much lower level of infiltration) was not associated with a pCR subsequently in LLABCs following NAC.

**Figure 2:**
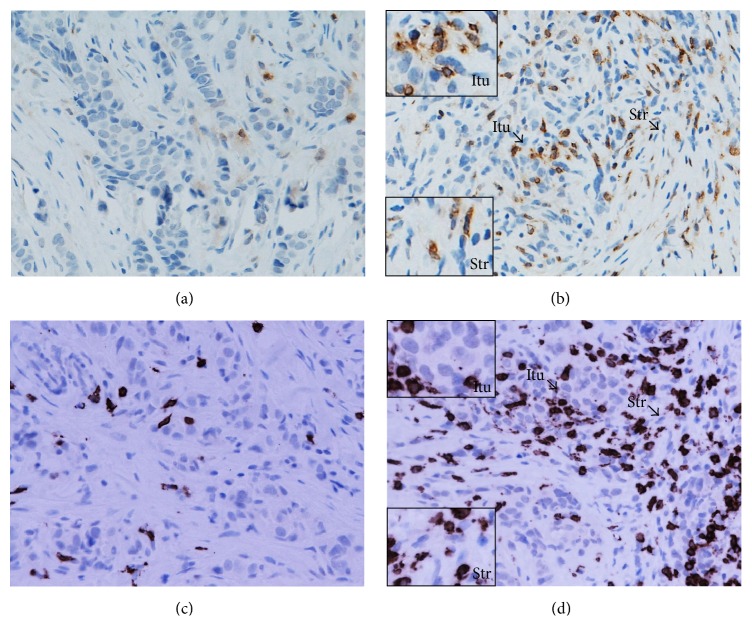
CD4^+^ (a, b) and CD8^+^ (c, d) T lymphocytes in the sections of LLABCs, using IHC staining, at 400x magnification. Briefly, heat-mediated antigen retrieval was performed using citrate buffer, pH 6 (20 mins). The sections were then incubated with MAbs to CD4 (Dako, M7310) at a 1 : 80 dilution for 30 mins at RT and MAbs to CD8 (Dako, M7103) at a 1 : 100 dilution for 30 mins at RT. Polymeric HRP-linker antibody conjugate was used as secondary antibody. DAB chromogen was used to visualize the staining. The sections were counterstained with haematoxylin. (a, c) Low level of CD4^+^ and CD8^+^T cell infiltration; (b, d) high level of CD4^+^ and CD8^+^T cell infiltration. The average number of brown membrane-stained cells, regardless of intensity, in contact with tumour cells or within tumour cell nests (Itu: intratumoural) and in the interstitial stroma (Str: stromal) per HPF was counted.

**Figure 3:**
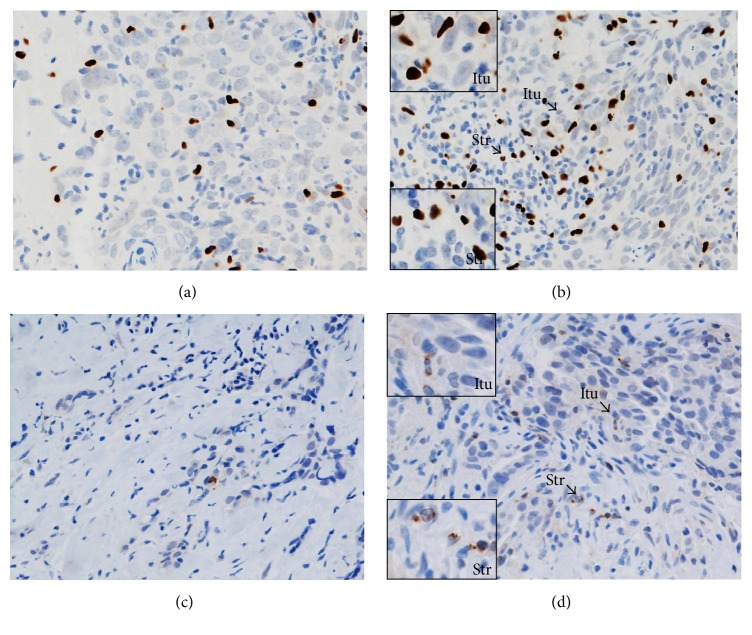
FOXP3^+^ (a, b) and CTLA-4^+^ (c, d) Tregs in the sections of LLABCs, using IHC staining, at 400x magnification. Briefly, heat-mediated antigen retrieval was performed using citrate buffer, pH 6 (20 mins). The sections were then incubated with MAbs to FOXP3 (Abcam, ab20034) at a concentration of 20 *μ*g/mL for 30 mins at RT and MAbs to CTLA-4 (Santa Cruz Bio, sc-376016) at a 1 : 300 dilution for 30 mins at RT. Polymeric HRP-linker antibody conjugate was used as secondary antibody. DAB chromogen was used to visualize the staining. The sections were counterstained with haematoxylin. (a, c) Low level of FOXP3^+^, CTLA-4^+^Treg infiltration; (b, d) high level of FOXP3^+^ and CTLA-4^+^Treg infiltration. The average number of brown nuclear-stained (FOXP3), membrane-stained (CTLA-4) cells, regardless of intensity, in contact with tumour cells or within tumour cell nests (Itu: intratumoural) and in the interstitial stroma (Str: stromal) per HPF was counted.

**Figure 4:**
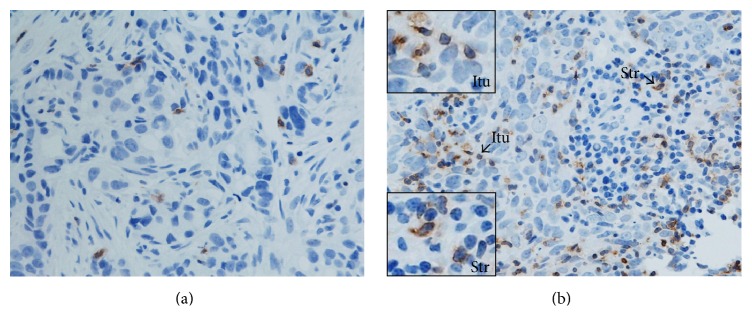
PD-1^+^ T cells in the sections of LLABCs, using IHC staining, at 400x magnification. Briefly, heat-mediated antigen retrieval was performed using citrate buffer, pH 6 (20 mins). The sections were then incubated with MAbs to PD-1 (Abcam, ab52587) at a 1 : 100 dilution for 30 mins at RT. Polymeric HRP-linker antibody conjugate was used as secondary antibody. DAB chromogen was used to visualize the staining. The sections were counterstained with haematoxylin. (a) Low level of PD-1^+^ T cell infiltration; (b) high level of PD-1^+^ T cell infiltration. The average number of brown membrane-stained cells, regardless of intensity, in contact with tumour cells or within tumour cell nests (Itu: intratumoural) and in the interstitial stroma (Str: stromal) per HPF was counted.

A prominent level of stromal infiltration by CD4^+^ and CD8^+^T cells was also associated with a pCR following NAC (*p* = 0.001 and *p* = 0.002, resp.). Stromal infiltration by CTLA-4^+^ T cells was similarly associated with a pCR (*p* = 0.041) but infiltration by FOXP3^+^and PD-1^+^T cells was not (Table 2) (Figures 2, 3, and 4).

### 3.3. Tumour-Infiltrating CD8^+^ : FOXP3^+^ T Cell Ratio: Significant Association with a Pathological Complete Response (PCR) following NAC

Table 3 documents the significant association between the tumour-infiltrating CD8^+^ : FOXP3^+^ T cell ratio and pathological response. A good pathological response (grades 5 and 4) was seen with intratumoural (*p* = 0.027) and stromal (*p* = 0.027) infiltration ratios. Similar and more significantly pronounced ratios were seen with intratumoural (7.40 versus 1.48, *p* = 0.002) and stromal (5.37 versus 1.67, *p* = 0.001) pCRs (Table 3). Thus the concurrent high level of CD8^+^ and low level of FOXP3^+^T cells are an important factor predisposing to a pCR with NAC in LLABCs.

### 3.4. Significant Correlations between TILs and Specific Lymphocyte Subsets and Grade of Pathological Response to NAC

Table 4 shows the significant correlations between tumour-infiltrating (intratumoural, stromal) lymphocytes, respectively (TILs: rho = 0.601, *p* < 0.001, and rho = 0.641, *p* < 0.001; CD4^+^ T cells (stroma): rho = 0.468, *p* = 0.006; CD8^+^ T cells: rho = 0.446, *p* = 0.009, and rho = 0.471, *p* = 0.006), and grade of pathological response (grade 1 (no pathological response) to grade 5 (pCR)) in the breast cancers following 8 cycles of NAC. Infiltrating intratumoural CD4^+^T cells failed to reach statistical significance (*p* = 0.073) (Table 4). There was also a significant correlation between the CD8^+^ : FOXP3^+^ T cell ratios intratumourally (rho = 0.511, *p* = 0.002) and stromally (rho = 0.484, *p* = 0.004) and the grade of response elicited in LLABCs with NAC (Table 4).

### 3.5. Infiltration by T Cell Subsets in LLABCs: Significant Subset Reductions (CD4^+^, FOXP3^+^, CTLA-4^+^, and PD-1^+^ T cells but Not CD8^+^ T Cells) following NAC

Various lymphocyte subsets (CD4^+^, CD8^+^, FOXP3^+^, CTLA-4^+^, and PD-1^+^ T cells) were documented infiltrating LLABCs (Table 5). The most prominent infiltration was by CD4^+^ and CD8^+^ T cells, there being a threefold increase for CD4^+^ T cells and a twofold increase for CD8^+^ T cells in the peritumoural stroma compared with the intratumoural (tumour cell nests) compartment (45.6 [6.8–242.0] versus 15.4 [2.6–171.0] and 43.6 [1.8–201.6] versus 20.2 [3.4–202.4]), respectively (Table 5) (Figure 2). There was a smaller but still prominent infiltration by FOXP3^+^ T cells, comparable in both compartments (15.9 [2.2–110.6] versus 14.8 [12.4–96.8]). CTLA-4^+^ T cells, on the other hand, were present in low numbers in both the stromal and intratumoural compartments (Table 5) (Figure 3). Similarly, the infiltration by PD-1^+^T cells was low, albeit there was a wide range of values in both the intra- and peritumoural compartments (Table 5) (Figure 4).

Eight cycles of NAC induced a substantial and significant reduction in various T lymphocyte subsets. There was a significant reduction in both the intratumoural (*p* = 0.010) and stromal (*p* = 0.006) CD4^+^ T cells. There was, however, no significant reduction in intratumoural (*p* = 0.278) or stromal (*p* = 0.326) CD8^+^ T cell infiltration after NAC, albeit there was some reduction in the level of infiltration in both compartments (Table 5). Following 8 cycles of NAC there was a significant reduction in both the intratumoural (*p* = 0.001) and stromal (*p* = 0.001) FOXP3^+^ T cells. There was also a significant reduction of stromal (*p* = 0.029) CTLA-4^+^ T cells. Although the intratumoural CTLA-4^+^ T cells were reduced as well, this just failed to reach statistical significance (*p* = 0.060) (Table 5). NAC significantly reduced intratumoural and stromal PD-1^+^T cells (*p* = 0.005 and *p* = 0.016, resp.) (Table 5). Thus 8 cycles of NAC significantly reduced all the above T lymphocyte subsets, apart from CD8^+^T cells, infiltrating LLABCs.

### 3.6. Significant Concurrent Reduction of FOXP3^+^ and CTLA-4^+^ T Cells in the Blood and Tumours in Women with LLABCs Undergoing NAC

There was a significant reduction of FOXP3^+^ T cells in the blood (% [*p* = 0.001], absolute numbers (AbNs) [*p* = 0.001]) and breast cancers (intratumoural [*p* = 0.001], stromal [*p* = 0.001]) following 8 cycles of NAC in the same cohort of 16 patients (Table 6). There was also a significant reduction of CTLA-4^+^ T cells in the blood (% [*p* = 0.017], AbNs [*p* = 0.001]) and breast cancers (stromal [*p* = 0.029]) following 8 cycles of NAC in the same cohort of 16 patients. The intratumoural infiltration just failed to reach statistical significance (*p* = 0.060) (Table 6). The reduction in the tumour was at least 10-fold for FOXP3^+^ and 4-fold for CTLA-4^+^. This was more pronounced than the reduction seen in blood (twofold for FOXP3^+^ and CTLA-4^+^ %).

The blood FOXP3^+^ and CTLA-4^+^ T cell results were from a much larger cohort of patients and have been published by us previously [21].

There was a positive correlation between post-NAC % of blood FOXP3^+^ T cells and post-NAC intratumoural FOXP3^+^ T cells (rho = 0.687, *p* = 0.003). There was also a nonsignificant trend for a correlation between pre-NAC AbNs of blood and post-NAC intratumoural FOXP3^+^ T cells (rho = 0.470, *p* = 0.066). There were no correlations demonstrated for CTLA-4^+^ T cells (see Additional Files 2 and 3).

### 3.7. FOXP3^+^/CTLA-4^+^ T Cell Profile (Blood, Tumour-Infiltrating) and Pathological Response to NAC

At diagnosis and prior to NAC, there were no significant differences in the levels of circulating (%, AbNs) and tumour-infiltrating T cells (FOXP3^+^, CTLA-4^+^) and the subsequent different NAC response groups (good pathological response versus poor pathological response and pCR versus non-pCR) (see Additional File 4).

After NAC, however, there was a significantly higher % of blood FOXP3^+^ T cells and significantly higher levels of intratumoural (tumour cell nests) FOXP3^+^ T cells in those women whose tumours had a poor pathological response to 8 cycles of NAC (*p* = 0.001 and *p* = 0.016, resp.) or failed to demonstrate a pCR (*p* = 0.007 and *p* < 0.001, resp.) (Table 7). In the case of CTLA-4^+^ T cells, higher blood levels of AbNs were significantly associated with a poor pathological response to 8 cycles of NAC (*p* = 0.008) (Table 7).

### 3.8. Cytokine (TH1, TH2, and TH17) Profiles in the Tumour Microenvironment: Association with NAC-Induced PCR

**Figure 5:**
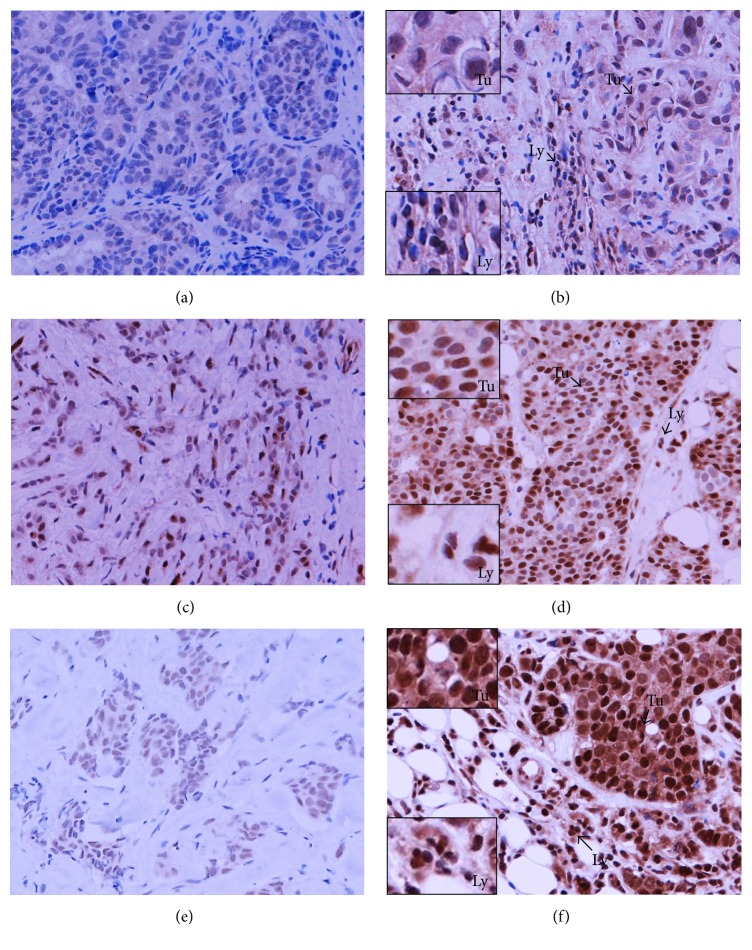
IL-1 (a, b), IL-2 (c, d), and IFN-*γ* (e, f) expression in the sections of LLABCs, using IHC staining, at 400x magnification. Briefly, heat-mediated antigen retrieval was performed using citrate buffer, pH 6 (20 mins). The sections were then incubated with MAbs to IL-1 (Abcam, ab8320) at a 1 : 150 dilution overnight at 4°C and MAbs to IL-2 (Abcam, ab92381) at a 1 : 500 dilution for 30 mins at RT and polyclonal Abs to IFN-*γ* (Abcam, ab9657) at a concentration of 4 *μ*g/mL for 30 mins at RT, respectively. Polymeric HRP-linker antibody conjugate was used as secondary antibody. DAB chromogen was used to visualize the staining. The sections were counterstained with haematoxylin. (a, c, e) Low level of expression; (b, d, f) high level of expression. The *H* score (% of positive cells (brown membrane/cytoplasmic-stained tumour and immune cells) × intensity of staining (1 to 3)) was used to assess the level of expression; low was ≤100 and high was >100. Scoring performed on whole tissue section (>10 HPFs); Tu: tumour and Ly: lymphocyte.

**Figure 6:**
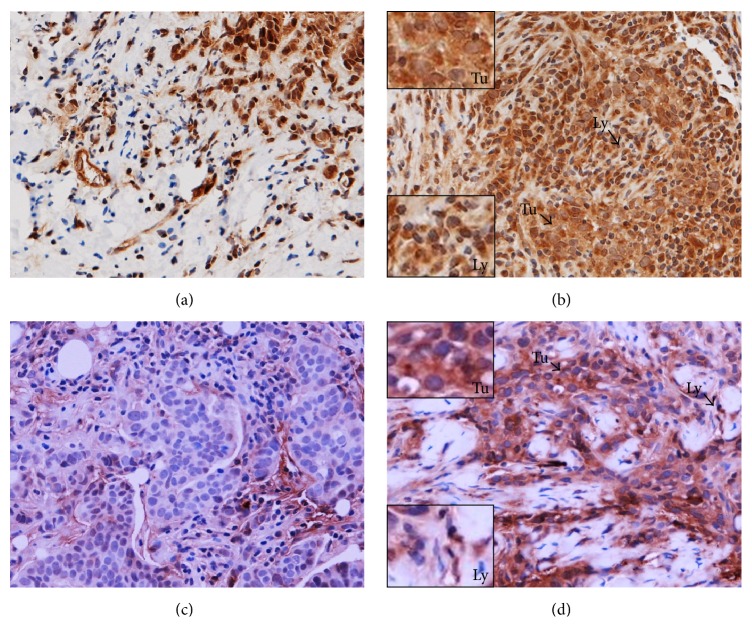
IL-4 (a, b) and IL-10 (c, d) expression in the sections of LLABCs, using IHC staining, at 400x magnification. Briefly, heat-mediated antigen retrieval was performed using citrate buffer, pH 6 (20 mins). The sections were then incubated with polyclonal Abs to IL-4 (Abcam, ab9622) at a concentration of 4 *μ*g/mL for 30 mins at RT and polyclonal Abs to IL-10 (Abcam, ab34843) at a 1 : 400 dilution for 30 mins at RT. Polymeric HRP-linker antibody conjugate was used as secondary antibody. DAB chromogen was used to visualize the staining. The sections were counterstained with haematoxylin. (a, c) Low level of expression; (b, d) high level of expression. The *H* score (% of positive cells (brown membrane/cytoplasmic-stained tumour and immune cells) × intensity of staining (1 to 3)) was used to assess the level of expression; low was ≤100 and high was >100. Scoring performed on whole tissue section (>10 HPFs); Tu: tumour and Ly: lymphocyte.

**Figure 7:**
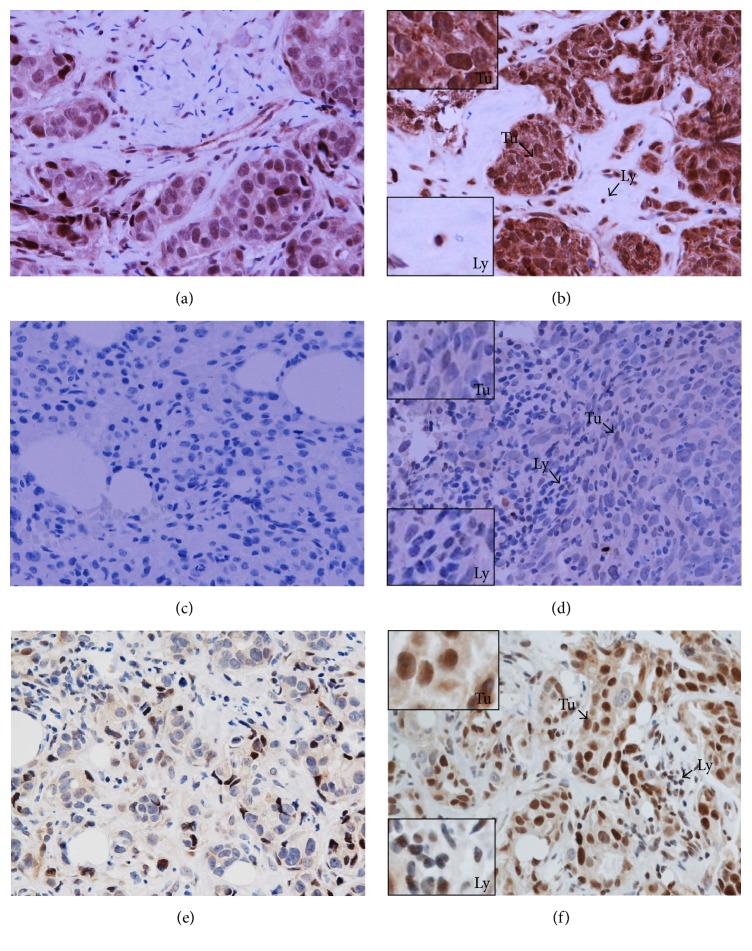
IL-17 (a, b), TGF-*β* (c, d), and PD-L1 (e, f) expression in the sections of LLABCs, using IHC staining, at 400x magnification. Briefly, heat-mediated antigen retrieval was performed using citrate buffer, pH 6 (20 mins). The sections were then incubated with polyclonal Abs to IL-17 (Abcam, ab9565) at a 1 : 100 dilution for 30 mins at RT, MAbs to TGF-*β* (Abcam, ab64715) at a concentration of 12 *μ*g/mL overnight at 4°C, and polyclonal Abs to PDL1 (Abcam, ab58810) at a concentration of 2.5 *μ*g/mL for 15 mins at RT, respectively. Polymeric HRP-linker antibody conjugate was used as secondary antibody. DAB chromogen was used to visualize the staining. The sections were counterstained with haematoxylin. (a, c, e) Low level of expression; (b, d, f) high level of expression. The *H* score (% of positive cells (brown membrane/cytoplasmic-stained tumour and immune cells) × intensity of staining (1 to 3)) was used to assess the level of expression; low was ≤100 and high was >100. Scoring performed on whole tissue section (>10 HPFs); Tu: tumour and Ly: lymphocyte.

Table 8 documents the expression of various cytokines in the tumour microenvironment in women with LLABCs, prior to and following 8 cycles of NAC. There was no significant association with a pCR following NAC and the expression* in situ* of Th1 (IL-2, IFN-*γ*) cytokines (Figure 5). There was, however, a significant association with a failed pCR following NAC and the expression* in situ* of the immunosuppressive Th2 cytokine IL-10 (*p* = 0.039) (Figure 6). Expression* in situ *of the Th17 cytokine IL-17 was also significantly associated with a poor pathological response and failure to achieve a pCR (*p* = 0.013) (Table 8) (Figure 7). There was a nonsignificant association between the* in situ *expression of the immunosuppressive cytokine TGF-*β* and a pCR in the breast cancer following NAC (*p* = 0.062) (Figure 7).

NAC had no significant effect on the expression* in situ* of the Th1 (IL-2, IFN-*γ*), Th2 (IL-10), and Th17 (IL-17) cytokines in the tumour microenvironment. The expression of the Th2 cytokine IL-4, however, was significantly altered following NAC (*p* = 0.016) (see Additional File 5). There was a high level of expression of IL-4 (87.5% (14 out of 16)) in the pre-NAC specimens. Following NAC, 43.8% (7 out of 16) of tumour samples showed alteration in the level of expression of IL-4. 50% (7 out of 14) of the tumour samples showing a high level of expression before NAC were altered to a low/negative level of expression of IL-4 after NAC. None of the cases studied (0%) changed to a high level of expression. A nonsignificant reduction of* in situ* IL-2 expression was also seen after NAC, being reduced from 11 out of 16 (68.8%) in pre-NAC specimens to 5 out of 16 (31.3%) in post-NAC specimens (*p* = 0.070).

### 3.9. Clinicopathological Characteristics and T Lymphocytic Subsets (CD4^+^, CD8^+^, and FOXP3^+^) Infiltrating LLABCs

Table 9 documents a range of clinical features, NAC regimens, and tumour characteristics of the patients studied. There was a significant association of T lymphocyte subsets (CD4^+^, CD8^+^, and FOXP3^+^) with tumour grade: infiltration by CD4^+^ and CD8^+^ T cells, intratumoural (*p* = 0.026 and *p* = 0.038, resp.) and stromal (*p* = 0.004 and *p* = 0.032, resp.), and stromal infiltration by FOXP3^+^ T cells (*p* = 0.018). High levels of tumour infiltration by these three T cell subsets were significantly associated with high grade (3) tumours. There was no obvious association with the other pathological and clinical parameters in the small patient (*n* = 33) sample.

Univariate analysis showed the following predictive factors for pCR: TILs (*p* = 0.001), tumour grade (*p* = 0.005), and oestrogen receptor (ER) status (*p* = 0.049) (Table 10). Multivariate analysis, however, showed that TILs were the only independent predictor of a pCR in the 33 patients studied with LLABCs undergoing NAC (Table 10).

## 4. Discussion

The presence of a high level of TILs in various human solid cancers, including breast cancer, has been shown to be a reliable prognostic indicator and associated with an improved clinical outcome [4, 6–8, 31, 32]. Studies of specific T cell subsets, however, have produced variable results in different tumour types [2].

The association of TILs and different lymphocyte subsets in breast cancer patients undergoing NAC and contributing to tumour cell death has generated clinical and scientific interest. Demaria et al. (2001) first showed that, following NAC with paclitaxel, the presence of TILs following treatment correlated with the pathological response elicited in the breast cancer to NAC [33]. Several studies have subsequently shown TILs to be important predictors of a pathological response, in particular a pCR [9, 11, 34]. In fact, Denkert et al. (2010) showed TILs to be an independent predictor for a pCR in women undergoing NAC [9]. Our study, with a much smaller cohort of patients, also showed high levels of TILs to be an independent predictive factor (multivariate analysis) for a pCR, a recognised surrogate marker of a good outcome in breast cancer following NAC [23, 24]. West et al. (2011) reported that the presence of TILs in breast cancer was a good predictor of a pCR in patients with ER −ve tumours who had received an anthracycline-based NAC regimen [35]. Ono et al. (2012) demonstrated that TILs correlated with response to NAC (anthracycline ± taxane-based) in triple −ve breast cancer [34]. Dieci et al. (2014) also demonstrated that high levels of TILs (stromal and intratumoural) in the residual breast tumour following NAC in triple −ve cancers were significantly associated with a better disease-free survival (DFS) and overall survival (OS) [29]. Lee et al. (2013) showed TILs to be associated with a better prognosis in axillary lymph node (ALN) +ve, ER −ve, and HER2 −ve subtypes following NAC (anthracycline ± taxane-based) [36]. In our study, the NAC regimen consisted of cyclophosphamide, the anthracycline doxorubicin, and the taxane docetaxel ± capecitabine. Sixty-seven percent of the tumour specimens studied, however, were ER +ve and only 9% were triple −ve [24]. We showed a significant correlation between high levels of TILs (intratumoural, stromal) and the subsequent pathological grade of response (5–1) in LLABCs after 8 cycles of NAC, a finding not previously reported.

Although the impact of TILs in breast cancer, with or without NAC, has been documented in a large cohort of patients, the contribution of the various TIL subsets is inadequately studied and data for several of the subsets is poorly documented. There is a paucity of published data regarding CD4^+^ T cells infiltrating breast tumours. Droesser et al. (2012) found that they were not a prognostic indicator [37]. Heys et al. (2012) reported low levels of CD4^+^ T cells to be significantly associated with a better response to NAC [38].

In a range of human solid cancers (colorectal, ovarian, oesophageal, lung, breast, and pancreas) the presence of high levels of tumour-infiltrating CD8^+^ T cells (and CD45RO^+^ memory T cells) was associated with a favourable prognosis [1, 4]. Mahmoud et al. (2011) documented CD8^+^T cells in tumour cell nests and stroma. High CD8^+^T cell counts were independently associated with longer breast cancer-specific survival [15]. Matkowski et al. (2009), however, showed that a high level of CD8^+^T cells in breast tumours was associated with high tumour grade, ER negativity, expression of HER2, metastatic spread to ALNs, and a poor prognosis [39]. A small number of studies have documented the relevance of tumour-infiltrating CD8^+^ T cells with NAC. Two studies found that high levels of CD8^+^ T cells in breast cancer were associated with a pCR following NAC [27]. In HER2 overexpressing breast cancers, a high CD8^+^ : FOXP3^+^ T cell ratio was associated with a pCR and an improved DFS and OS [40].

Our study demonstrated infiltration by both CD4^+^and CD8^+^T cells, with a predominance in the peritumoural stroma compared with tumour cell nests. This profile and compartmentalisation of effector T cells in breast cancer are not well characterised in the literature. Degnim et al. (2014) documented the pattern of infiltration by CD4^+^ and CD8^+^ T cells in normal human breast lobules [41]. CD4^+^ T cells were comparable (median, interquartile range) with the intratumoural levels documented in our patients. CD8^+^ T cells, however, were more prominent and 2.5-fold higher than the intratumoural levels documented in our patients. Thus, in breast cancer there is a reduction of the normal CD8^+^: CD4^+^ T cell ratio due to lower levels of CD8^+^ T cell infiltration. Following NAC there was a significant reduction in both the intratumoural and stromal CD4^+^ T cells but not CD8^+^ T cells, albeit there was some reduction in CD8^+^ T cell levels.

In our study high levels of CD4^+^ and CD8^+^ T cells, intratumourally and stromally, in LLABCs were associated with a subsequent pCR following NAC. These findings are in agreement with recently published data [27, 42–44]. We also established that a high CD8^+^ : FOXP3^+^ T cell ratio in LLABCs prior to NAC was associated with a subsequent pCR. Ladoire et al. (2011) documented similar findings in a HER2 overexpressing breast cancer subset. The majority of the tumours in our study, however, (as in breast cancer in general), were HER2 −ve. Our study also demonstrated a significant correlation between tumour-infiltrating CD4^+^and CD8^+^T cells, CD8^+^ : FOXP3^+^ T cell ratio and the pathological grade of response (5–1) elicited with NAC. To the best of our knowledge, such findings have not been previously published. Moreover, we have recently documented a significant correlation between high levels of stromal infiltration by natural killer (NK) cells and pathological grade of response in LLABCs [20]. Thus, our findings suggest that various adaptive and innate lymphocyte subsets appear to play an important role in facilitating an effective anticancer response associated with NAC, in women with LLABCs. Functional assays need to be carried out on isolated T cell subsets to define more precisely these roles.

CD4^+^ T cells include different Th cell subsets (Th1, Th2, and Th17) secreting a wide range of pro- and anti-inflammatory cytokines (Il-2, IFN-*ϒ*, IL-4, IL-5, IL-10, and IL-17), as well as natural and inducible CD4^+^CD25^+^ FOXP3^+^ Tregs. These subsets show a degree of plasticity (Th17 cells secreting the Th1 cytokine IFN-*ϒ*; transformation of Tregs into Th1 and Th17 subsets) [45]. This complex profile makes it difficult to attribute precisely the contribution of each subset or combination of CD4^+^ Th cell subsets to a pCR with NAC. The lack of association with pCR of FOXP3^+^ T cells (putative Tregs) suggests an important role for the Th subsets. CD8^+^ T cells also consist of different subsets, namely, naive, memory, and activated CD8^+^ cytotoxic T lymphocytes (CTLs). CD8^+^ T suppressor cells, lacking expression of CD28 but expressing CD122 and FOXP3, have also been described. This is a highly restricted and weak suppressor cell subset [46].

Interest has focused on the possible contribution of FOXP3^+^TILs to prognosis and pathological responses in breast cancer induced by NAC and is a matter of continuing debate [13, 28, 40, 47]. Bates et al. (2006) studied normal breast tissue (reduction mammoplasties) and found very low levels of infiltration by FOXP3^+^ T cells [48]. High levels of FOXP3^+^ T cells in breast tumours have been reported in both ductal carcinoma* in situ* (DCIS) and in much higher levels in invasive breast cancer [14, 48, 49]. Our study showed a 45-fold higher level of FOXP3^+^ T cells in the LLABC specimens (median, interquartile range), compared with normal breast tissue [48]. High levels of FOXP3^+^T cells have been found to be significantly increased in HER2 +ve breast cancers [13, 50]. In our study, FOXP3^+^T cells were also prominent in HER2 −ve cancers (major phenotype in breast cancer).

Tregs (FOXP3^+^) play an important role in the control of autoimmunity, maintenance of transplantation tolerance and suppression of anticancer immune responses. FOXP3 is a transcription factor required for the generation of CD4^+^ CD25^+^ Tregs and is a key marker for identifying such cells. Tregs in peripheral tissues are a mixture of natural and induced FOXP3^+^ Tregs. Induced FOXP3^+^ Tregs have a more heterogeneous phenotype (some cells lack CD25) and are induced by TGF-*β* and IL-10 [51]. In the breast cancer tissue sections studied, there was* in situ *expression of IL-10 and TGF-*β* and therefore the likely presence of induced FOXP3^+^ Tregs. It was not possible, however, to distinguish between the two Treg types. Both, on the other hand, contribute to inhibition of immune responses. The contribution by CD8^+^FOXP3^+^ Tregs is likely to be minimal as they are a small subset with weak immune suppressive activity [46].

Tregs are generated in the early phase of the adaptive immune response and IL-2 is central to their development and survival. They suppress the function of a wide range of immune cells (CD4^+^ and CD8^+^ T cells, NK and NK T cells, and dendritic cells (DCs)) [52, 53]. As a substantial number of human CD4^+^ T cells transiently express FOXP3^+^ during activation but not necessarily acquisition of regulatory function caution has been expressed about its uniqueness as a marker for Tregs [51].

Increased levels of FOXP3^+^ Tregs have been documented in blood, lymph nodes, and infiltrating various human tumours [2, 14, 21, 54–56]. In many, a high level of FOXP3^+^ T cell infiltration was shown to be associated with an unfavourable clinical outcome [2, 14, 48]. In some cancers (colorectal, ovarian, bladder, head, and neck) high levels of tumour-infiltrating FOXP3^+^ T cells were found to be associated with an improved prognosis [2, 57]. Bates et al. (2006) reported that the presence of FOXP3^+^T cells identified breast cancer patients at high risk of relapse [48]. Gobert et al. (2009) found regulatory T cells to be selectively activated in lymphoid infiltrates in breast cancers, leading to a poor prognosis [58]. Demir et al. (2013) stated that intratumoural FOXP3^+^T cells were prognostic factors in LLABCs [28]. Mahmoud et al. (2011), however, did not demonstrate any relationship to clinical outcome with tumour-infiltrating FOXP3^+^ Tregs in breast cancers [16]. Paradoxically, high levels of FOXP3^+^Tregs in ER −ve breast cancers (less common type of breast cancer) were shown to be associated with a good clinical outcome [59].

Oda et al. (2012) documented that high levels of tumour FOX3^+^ T cells prior to NAC were associated with high pCR rates [27]. In a cohort of patients with HER2 +ve cancers there was a better OS and DFS if the breast cancer cells themselves expressed FOXP3^+^, possibly acting as a tumour suppressor gene [60]. In our study, the majority of breast samples were HER2 −ve and in only one specimen was FOXP3^+^expressed in the breast cancer cells. Lui et al. (2012) reported that decreased stromal FOXP3^+^ Tregs after NAC were associated with a pCR, whilst intratumoural reduction after NAC was an independent prognostic predictor of OS [47]. High levels of FOXP3^+^ Treg infiltration after NAC, however, correlated with enhanced rates of pCR in another study [28]. Our results are in agreement with the published findings regarding CD8^+^ : FOXP3^+^ T cell profiles and the post-NAC reduction of FOXP3^+^ T cells and pCR (surrogate marker of improved survival). Our findings, however, do not agree with the data reporting a beneficial response to NAC with high levels of FOXP3^+^ cell infiltration before and after NAC. The reasons for this discrepancy are not clear.

CTLA-4 (CD152) is a coinhibitory receptor molecule expressed on activated T cells and Tregs that negatively regulates T cell interaction with B7-1 (CD80)/B7-2 (CD86) ligand binding sites competing with CD28 which upregulates T cell activation [61, 62]. There is little expression on inactive or naive Tregs [63, 64]. CTLA-4 inhibits the interaction of CD28 receptors on CD4^+^and CD8^+^T cells with CD80/86 ligands on DCs and reduces IL-2 production, IL-2 receptor expression, and cell cycle progression of activated T lymphocytes, resulting in inhibition of activated DCs and generation of CD4^+^Th subsets and CD8^+^ CTLs [65, 66]. Thus CTLA-4 is an important immune checkpoint inhibitor of both CD4^+^ and CD8^+^ T effector cells preventing inappropriate and prolonged T cell activation and resultant tissue damage. In breast cancer there is increased expression of CTLA-4, compared with normal breast tissue [66]. Increased mRNA levels of CTLA-4 were shown to be associated with ALN metastases and more advanced tumour stage [66, 67]. We had previously demonstrated high levels of CTLA-4^+^ cells in the blood of women with LLABCs [21]. In our current study there was a wide range of levels of CTLA-4^+^cells infiltrating the LLABCs but overall, the levels were low.

We demonstrated a significant reduction of FOXP3^+^(intratumoural, stromal) and CTLA-4^+^T cells (stromal) in tumours following 8 cycles of NAC. The FOXP3 findings are in agreement with published data [28, 44, 47]. The CTLA-4^+^ T cell findings have not been previously reported. We also showed a concurrent significant reduction of Tregs (FOXP3^+^, CTLA-4^+^) in the blood of the same cohort of patients [21]. There was, moreover, a positive correlation between the post-NAC % of blood FOXP3^+^Tregs and post-NAC intratumoural infiltration by FOXP3^+^T cells. Thus, the significant reduction in the circulating levels of Tregs in women with LLABCs undergoing NAC was associated with a substantial and significant concomitant reduction of FOXP3^+^ and CTLA-4^+^T cells infiltrating the breast tumours. After NAC there was a significantly higher % of blood FOXP3^+^Tregs and significantly higher level of intratumoural (tumour cell nests) FOXP3^+^T cells in patients whose tumours had a poor pathological response and failed to demonstrate a pCR. To the best of our knowledge, these various findings have not been previously published. Our results highlight the importance of regulatory suppressor mechanisms in the circulation and tumour environment in inducing immune-mediated tumour cell death with NAC.

PD-1 (CD279) is a transmembrane receptor and a member of the CD28 family and is expressed on activated T cells and other lymphocytes (Tregs, NK cells, and B cells) [68–70]. When interacting with PD-L1 and PD-L2 in a coinhibitory pathway in peripheral tissues it dampens down activated T cells (cytotoxic activity, proliferation, and cytokine production) maintaining peripheral T cell tolerance and preventing autoimmunity [71]. The PD-1 pathway is one of the immune checkpoints exploited by cancer cells to escape anticancer immune defenses [72]. PD-L1 is expressed on different lymphoid cells, is upregulated in various normal cells in inflammation, and is expressed in many human cancers. It has been shown to correlate with tumour size, grade, metastatic spread, and reduced levels of tumour-infiltrating CD8^+^ T cells [73–75]. High levels of PD-1^+^ cells have been shown to have a significant correlation with reduced patient survival [76]. In our study, although there was a wide range in both the intra- and peritumoural stromal compartments, the infiltration in general was low. A significant reduction of both intratumoural and stromal infiltration by PD-1^+^T lymphocytes was seen following 8 cycles of NAC. The level of infiltration in LLABCs, however, was not associated with a subsequent pCR following NAC. There is a lack of data in the literature about the effect of NAC on the PD-1^+^ T cell subsets infiltrating LLABCs. We believe this to be a newly reported finding.

In various human cancers malignant cells and host infiltrating cells express and secrete a range of Th1, Th2, and Th17 cytokines (IL-1*β*, IL-2, IL-4, IL-6, IL-10, IL-17, and IFN-*γ*) and TGF-*β*. These cytokines modulate and suppress the* in situ* anticancer immune responses, enhancing tumour cell growth and progression, and propensity to metastasize [77–83]. In our study the semiquantitative method used did not discriminate between the tumour-infiltrating immune and inflammatory cells and the malignant cells nor quantify precisely the contribution of the various host immune and inflammatory cells to the cytokine levels in the tumour microenvironment.

In the tumour microenvironment Th1, Th2, and Th17 cytokines, as well as TGF-*β*, play an important role in modulating* in situ* innate and adaptive immune mechanisms [84]. The Th1 cytokines IL-2 and IFN-*ϒ* enhance CTL-and NK cell-mediated regression of cancer cells. IFN-*ϒ* can either promote or suppress Treg activity depending on the cytokine environment. IL-2 also has a key role in controlling Treg function in the periphery [51]. The Th2 cytokines IL-4 and IL-10 suppress the generation of CTLs and Th1 cells and recruit tumour entry of Tregs [1, 53]. Moreover, IL-4 has been shown to both increase and inhibit Treg function. It can enhance FOXP3 expression and suppressor activity of Tregs and conversely can inhibit TGF-*β* induced Treg development [85, 86]. Th1 and Th2 cytokine expression in tumours has a variable effect on patient outcomes in a range of human cancers, including breast cancer [2]. The role of IL-17 is not well defined. Some animal studies suggest it promotes tumour growth and angiogenesis [87, 88]. Yamazaki et al. (2008) have shown that IL-17 promotes the recruitment of Tregs to sites of IL-17 mediated inflammation [89]. Others have suggested an increased generation of CTLs and an enhanced tumour rejection [90, 91]. Contradictory results have been demonstrated in a range of human tumours, including breast cancer [2]. In one study in breast cancer, the level of Th17 cells was shown to be increased and associated with an improved prognosis [81]. TGF-*β* expression is usually upregulated in human cancers. It induces production of FOXP3^+^ Tregs and has strong immunosuppressive effects, inhibiting the generation and activity of innate (DCs, NK cells) and adaptive (CD4^+^ and CD8^+^ T cells) immunity [53, 84]. TGF-*β* can promote an epithelial to mesenchymal transition, resulting in enhanced tumour cell mobility, local invasion, and formation of metastases [92]. An inflammatory environment, not infrequent in tumours, can induce the transformation of FOXP3^+^ Tregs into FOXP3^−^ effector cells producing IFN-*ϒ* [93]. IL-6 can also induce FOXP3^+^ Treg loss and transformation to a Th17 phenotype and function [94]. This is further evidence of the plasticity of the different CD4^+^ T cell effector-regulator subsets. The interplay between the different T cell profiles in human cancers is complex, the outcomes variable, and in need of further careful study.

The effect of NAC on Th1, Th2, and TH17 cytokine production in tumours is poorly documented. In our study, pre-NAC levels of expression were not associated with a pCR following NAC. IL-4 was significantly reduced in the tumour microenvironment following NAC. A similar but nonsignificant trend was seen with the* in situ *expression of IL-2 (*p* = 0.070). Post-NAC expression of IL-10 and IL-17, however, showed a significant association with failure to achieve a pCR. There was a similar trend for the* in situ* presence of TGF-*β* (*p* = 0.062). These various findings with NAC have not previously been reported.

High tumour grade is known to be associated with a NAC-induced pCR in breast cancer. High tumour grade was shown to be significantly associated with tumour infiltration (intratumoural, stromal) by CD4^+^ and CD8^+^ T cells and stromal FOXP3^+^ T cells, which may have contributed to the NAC-induced pCR. There was no significant association with any of the clinical or other pathological parameters studied. This may be due to the relatively small number of specimens studied. In a multivariate analysis, a high TIL level was a significant independent predictor of a pCR with NAC and is in agreement with published data.

Most chemotherapeutic agents inhibit aspects of innate and adaptive immunity. Some, however, can enhance anticancer immunity and activate immune-mediated tumour cell death [19, 95–97]. Chemotherapy can induce cancer cell stress/damage resulting in the release of “danger” signals (e.g., heat shock proteins) and immunogenic tumour-associated antigens (TAAs). The former activate innate immune cells, whilst the latter are taken up by DCs leading to the release of proinflammatory cytokines and the generation of anticancer CTL responses. Anthracyclines, in particular, induce tumour cell damage and exposure of calreticulin and other endoplasmic reticulum proteins, secretion of ATP, and release of the high-mobility group box 1 (HMGB1) molecules. These interact with receptors on DCs, stimulating uptake and presentation of TAAs to naive T cells [18, 98–100].

The NAC combination (anthracycline, cyclophosphamide, and taxane ± capecitabine) used in our trial is known to have immunomodulatory effects. Doxorubicin has been shown to enhance the generation of antigen-specific CD8^+^ T cells and promote tumour infiltration by activated IFN-*γ* producing CD8^+^ T cells [69, 101].* In vitro*, doxorubicin increased antigen-specific CD4^+^ Th1 responses by inducing expression of CD40L and 4-1BB on CD4^+^ T cells [69]. Cyclophosphamide inhibits the generation and function of FOXP3^+^ Tregs in humans with various cancers [97, 102]. Taxanes have been shown to have immune stimulatory effects against tumours [95, 103]. In patients with advanced breast cancer, docetaxel therapy was associated with an increase in serum IFN-*γ*, IL-2, and IL-6 levels and enhancement of circulating NK cell activity [95]. Capecitabine is enzymatically converted to 5-fluorouracil (5-FU) on ingestion. 5-FU is known to increase the expression of TAAs on tumour cells and to enhance antibody-dependent cell-mediated cytotoxicity [104]. In mice, 5-FU induced depletion of immunosuppressive myeloid-derived suppressor cells and enhanced production of IFN-*γ* by tumour-infiltrating CD8^+^ T cells [105].

The NAC combination used in our study differentially preserved the tumour-infiltrating CD8^+^ T cell population but significantly reduced both the circulating and tumour-infiltrating FOXP3^+^, CTLA-4^+^ (stromal), and immune checkpoint PD-1^+^ T cells, thereby preventing the secretion of inhibitory cytokines (IL-4, IL-10, and TGF-*β*) and disrupting the PD-1/PD-L1 pathway. The restoration of immune anticancer effector mechanisms is likely to lead to an enhancement of immune-mediated tumour cell death. Moreover, the significant correlation of high CD8^+^ T cells and CD8^+^ : FOXP3^+^ T cell ratio with pCR (and hence DFS and OS) suggests a close association between high levels of CD8^+^ T cells/CTLs and the concomitant depletion of Tregs. Dysfunctional CD8^+^ T cell responses as a result of excessive and prolonged stimulation and continuous inappropriate signal activation result in T cell exhaustion and loss of effector and memory function. This persists even after removal of Tregs [106]. The close interrelationship between a pCR in LLABCs and the concomitant immune changes induced by NAC suggests that immune-mediated cell death may be a crucial component of NAC-associated tumour cell destruction and removal. A better understanding of this complex relationship in human cancer, in particular, the factors preventing optimal delivery of immune-mediated tumour cell death, is essential for devising more effective chemotherapeutic strategies in the management of cancer.

## 5. Conclusions 

Our study has confirmed previously published findings and documented novel findings, further establishing that the immune microenvironment is a key contributing factor in achieving a better pathological response with NAC. The level of TILs and CD4^+^ and CD8^+^ T cell subsets in LLABCs, which were well demonstrated with the IHC techniques used, could be clinically useful to further define women with LLABCs who may benefit from NAC. These biological markers can be readily determined from histopathological examination of breast tumour biopsies (using H&E and IHC) before commencing therapy. They may supplement other clinical parameters in establishing optimal treatment, as well as prognostic prediction, for individual women with LLABCs suitable for NAC.

## Supplementary Material

Additional file 1: documents the impact of NAC on the levels of TILs between pre- and post-NAC samples. The levels of both intra-tumoural and stromal TILs were not significantly altered, when pre-NAC samples were compared with post-NAC samples. Five out of 16 patients with a high level of TILs subsequently had a low level after NAC, whilst 1 out of 16 with a low level of TILs had a higher level post-NAC (*p* = 0.219). Additional file 2: prior to NAC, there was no significant correlation between the levels of circulating Tregs and those in the tumour microenvironment. Following NAC, however, there was a significant positive correlation between the % of circulating and intra-tumoural FOXP3^⁺^ Tregs. [Correlation Coefficient (rho) 0.687, *p* = 0.003]. Additional file 3: shows no significant correlation between circulating and tumour-infiltrating CTLA-4^⁺^ Tregs. Additional file 4: similar to pre-NAC tumour-infiltrating Tregs (FOXP3^⁺^ and CTLA-4^⁺^), the levels of pre-NAC circulating Tregs (AbNs and %) were not significantly different in any of the NAC response groups (GPR versus PPR and pCR versus non pCR, *p* > 0.05). Additional file 5: illustrates the effect of NAC on the expression of cytokines and PD-L1 in breast cancers. There was no significant difference between pre- and post-NAC expression (*p* > 0.05) except for IL-4. The expression of IL-4 following NAC was significantly reduced (*p* = 0.016); in 43.8% (7 out of 16) from high to low and in no case was this reversed.

## Figures and Tables

**Table 1:** Levels of tumour-infiltrating lymphocytes (TILs) in women with LLABCs^(1)^ and subsequent pathological complete response following NAC^(2)^.

TILs	Groups	Low infiltration (*n*)	High infiltration (*n*)	Pearson chi-square value (PCR^(3)^ versus non-PCR)	*p* value
Intratumoural	Pathological complete response (PCR, *n* = 16)	6	10	11.890	0.001^*∗*^
	Nonpathological complete response (non-PCR, *n* = 17)	16	1		

Stromal	Pathological complete response (PCR, *n* = 16)	3	13	16.051	<0.001^*∗*^
	Nonpathological complete response (non-PCR, *n* = 17)	15	2		

LPBC^(4)^	Pathological complete response (PCR, *n* = 16)	3	13	13.350	<0.001^*∗*^
	Nonpathological complete response (non-PCR, *n* = 17)	14	3		

^(1)^LLABCs: large and locally advanced breast cancers; ^(2)^NAC: neoadjuvant chemotherapy; ^(3)^PCR (grade 5): no residual invasive disease; ^(4)^LPBC: lymphocyte-predominant breast cancer; ^*∗*^statistically significant.

**Table 2:** Levels of tumour-infiltrating T cell subsets in women with LLABCs^(1)^ and subsequent pathological complete response following NAC^(2)^.

T cell subsets	Groups	Intratumoural Median (range)^(3)^	*p* value^(4)^ (PCR^(5)^ versus non-PCR)	Stromal Median (range)^(3)^	*p* value^(4)^ (PCR versus non-PCR)
CD4^+^	Pathological complete response (PCR, *n* = 16)	45.2 (1.6–171.0)	0.023^*∗*^	43.4 (1.0–242.0)	0.001^*∗*^
	Nonpathological complete response (non-PCR, *n* = 17)	5.8 (0.6–166.2)		10.4 (1.0–113.0)	

CD8^+^	Pathological complete response (PCR, *n* = 16)	40.6 (5.2–202.4)	0.008^*∗*^	75.5 (5.6–201.6)	0.002^*∗*^
	Nonpathological complete response (non-PCR, *n* = 17)	12.8 (0.4–99.2)		12.2 (1.8–110.0)	

FOXP3^+^	Pathological complete response (PCR, *n* = 16)	6.3 (0.4–96.8)	0.958	12.5 (0.8–110.6)	0.363
	Nonpathological complete response (non-PCR, *n* = 17)	5.4 (0.8–45.6)		10.8 (0.8–44.8)	

CTLA-4^+^	Pathological complete response (PCR, *n* = 16)	0.5 (0.0–4.0)	0.068	1.4 (0.0–10.0)	0.041^*∗*^
	Nonpathological complete response (non-PCR, *n* = 17)	0.4 (0.0–2.2)		0.4 (0.0–2.2)	

PD-1^+^	Pathological complete response (PCR, *n* = 6)	2.6 (0.0–57.4)	0.118	1.9 (0.4–81.2)	0.093
	Nonpathological complete response (non-PCR, *n* = 10)	0.5 (0.0–3.2)		0.9 (0.0–3.6)	

^(1)^LLABCs: large and locally advanced breast cancers; ^(2)^NAC: neoadjuvant chemotherapy; ^(3)^average cell count per 400x high-power field (core biopsies of breast cancers); ^(4)^Mann–Whitney *U* test; ^(5)^PCR (grade 5): no residual invasive disease; ^*∗*^statistically significant.

**Table 3:** Tumour-infiltrating CD8^+^ : FOXP3^+^ T cell ratio in LLABCs^(1)^ and subsequent pathological response to NAC^(2)^.

T cell subsets (*n* = 33)	Groups	Pre-NAC intratumoural Median (range)^(3)^	*p* value^(4)^ (GPR versus PRR, PCR versus non-PCR)	Pre-NAC stromal Median (range)^(3)^	*p* value^(4)^ (GPR versus PRR, PCR versus non-PCR)
CD8^+^ : FOXP3^+^ T cell ratio	Good pathological response (GPR, *n* = 21)^(5)^	3.26 (0.18–45.00)	0.027^*∗*^	4.67 (0.53–23.29)	0.027^*∗*^
	Poor pathological response (PPR, *n* = 12)^(6)^	1.37 (0.67–6.04)		1.81 (0.10–6.78)	
	Pathological complete response (PCR, *n* = 16)^(7)^	7.40 (0.27–45.00)	0.002^*∗*^	5.37 (1.08–23.29)	0.001^*∗*^
	Nonpathological complete response (non-PCR, *n* = 17)	1.48 (0.18–6.04)		1.67 (0.10–6.78)	

^(1)^LLABCs: large and locally advanced breast cancers; ^(2)^NAC: neoadjuvant chemotherapy; ^(3)^CD8^+^ T cell/FOXP3^+^ Treg ratio; ^(4)^Mann–Whitney *U* test; ^(5)^GPR (good pathological response, grades 5 and 4): no residual invasive disease, >90% loss of invasive disease, respectively; ^(6)^PPR (poor pathological response, grades 3, 2, and 1): 30–90% loss of invasive disease, <30% loss of invasive disease, and no loss of tumour cells, respectively; ^(7)^PCR (pathological complete response, grade 5); ^*∗*^statistically significant.

**Table 4:** Correlations between tumour-infiltrating lymphocytes (TILs) and specific T cell subsets and grade of pathological response to NAC^(1)^ (Spearman's correlation coefficient (rho)) in women with LLABCs^(2)^.

Lymphocytes (*n* = 33)	Groups	Grade of pathological response^(3)^	
		Correlation coefficient	*p* value (2-tailed)
TILs	Intratumoural infiltration	0.601	<0.001^*∗*^
	Stromal infiltration	0.641	<0.001^*∗*^

CD4^+^ T cells	Intratumoural infiltration	0.316	0.073
	Stromal infiltration	0.468	0.006^*∗*^

CD8^+^ T cells	Intratumoural infiltration	0.446	0.009^*∗*^
	Stromal infiltration	0.471	0.006^*∗*^

CD8^+^: FOXP3^+^ T cell ratio	Intratumoural infiltration	0.511	0.002^*∗*^
	Stromal infiltration	0.484	0.004^*∗*^

^(1)^NAC: neoadjuvant chemotherapy; ^(2)^LLABCs: large and locally advanced breast cancers; ^(3)^pathological responses were graded from grades 1 to 5 (grade 1 (no loss of tumour cells), grade 2 (<30% loss of invasive disease), grade 3 (30–90% loss of invasive disease), grade 4 (>90% loss of invasive disease), and grade 5 (complete pathological response, no residual invasive disease)); ^*∗*^statistically significant.

**Table 5:** T cell subsets infiltrating tumours (intratumoural, stromal) in women with LLABCs^(1)^: significant reduction with NAC^(2)^.

T cell subsets (*n* = 16)	Groups	Pre-NAC Median (range)^(3)^	Post-NAC Median (range)^(3)^	*p* value^(4)^ Pre-versus post-NAC
CD4^+^	Intratumoural infiltration	15.4 (2.6–171.0)	3.0 (0.0–71.6)	0.010^*∗*^
	Stromal infiltration	45.6 (6.8–242.0)	6.3 (1.2–236.0)	0.006^*∗*^

CD8^+^	Intratumoural infiltration	20.2 (3.4–202.4)	10.3 (0.0–83.6)	0.278
	Stromal infiltration	43.6 (1.8–201.6)	27.1 (1.6–144.6)	0.326

FOXP3^+^	Intratumoural infiltration	14.8 (2.4–96.8)	0.7 (0.0–22.2)	0.001^*∗*^
	Stromal Infiltration	15.9 (2.2–110.6)	1.4 (0.4–28.4)	0.001^*∗*^

CTLA-4^+^	Intratumoural infiltration	0.4 (0.0–4.0)	0.1 (0.0–1.2)	0.060
	Stromal infiltration	0.6 (0.2–10.0)	0.1 (0.0–5.2)	0.029^*∗*^

PD-1^+^	Intratumoural infiltration	0.7 (0.0–57.4)	0.0 (0.0–0.6)	0.005^*∗*^
	Stromal infiltration	1.5 (0.0–81.2)	0.0 (0.0–4.0)	0.016^*∗*^

^(1)^LLABCs: large and locally advanced breast cancers; ^(2)^NAC: neoadjuvant chemotherapy; ^(3)^average cell count per 400x high-power field; ^(4)^Wilcoxon signed rank test; ^*∗*^statistically significant.

**Table 6:** Blood^(1)^ and tumour-infiltrating FOXP3^+^ and CTLA-4^+^ T cells in women with LLABCs^(2)^: significant reduction with NAC^(3)^.

T cell subsets (*n* = 16)	Groups	Pre-NAC Median (range)^(4)^	Post-NAC Median (range)	*p* value^(5)^ Pre- versus post-NAC
FOXP3^+^	Intratumoural Infiltrating	14.8 (2.4–96.8)	0.7 (0–22.2)	0.001^*∗*^
	Stromal Infiltrating	15.9 (2.2–110.6)	1.4 (0.4–28.4)	0.001^*∗*^
	% Circulating	1.54 (0.62–3.40)	0.81 (0.25–1.85)	0.001^*∗*^
	AbN circulating^(6)^	170 (107–427)	159 (35–230)	0.001^*∗*^

CTLA-4^+^	Intratumoural Infiltrating	0.4 (0.0–4.0)	0.1 (0.0–1.2)	0.060
	Stromal infiltrating	0.6 (0.2–10.0)	0.1 (0.0–5.2)	0.029^*∗*^
	% circulating	1.31 (0.05–3.24)	0.72 (0.10–1.71)	0.017^*∗*^
	AbN circulating	15 (5–19)	6 (2–15)	0.001^*∗*^

^(1)^Blood: data previously published (Verma et al., 2013 [21]); ^(2)^LLABCs: large and locally advanced breast cancers; ^(3)^NAC: neoadjuvant chemotherapy; ^(4)^average cell count per 400x high-power field; ^(5)^Wilcoxon signed rank test; ^(6)^AbN: absolute number (cells/mm^3^); ^*∗*^statistically significant.

**Table 7:** Blood^(1)^ and tumour-infiltrating FOXP3^+^ and CTLA-4^+^ T cells (post-NAC) in women with LLABCs^(2)^ and pathological response elicited in tumours following NAC^(3)^.

T cell subsets	Groups (*n* = 16)	Intratumoural Median (range)^(4)^	*p* value	Stromal Median (range)	*p* value	% blood Median (range)	*p* value	AbN blood Median (range)^(5)^	*p* value^(6)^
FOXP3^+^	GPR (*n* = 9)^(7)^	0.0 (0.0–2.4)	0.016^*∗*^	0.8 (0.4–7.4)	0.252	0.53 (0.25–0.90)	0.001^*∗*^	166 (35–230)	0.470
	PPR (*n* = 7)^(8)^	2.2 (0.6–22.2)		1.4 (1.0–28.4)		1.18 (0.80–1.85)		157 (118–168)	
	PCR (*n* = 6)^(9)^	0.0 (0.0–0.0)	<0.001^*∗*^	1.3 (0.4–7.4)	0.635	0.35 (0.25–0.90)	0.007^*∗*^	173 (49–230)	0.313
	Non PCR (*n* = 10)	1.8 (0.6–22.2)		1.4 (0.4–28.4)		1.15 (0.53–1.85)		158 (35–177)	

CTLA-4^+^	GPR (*n* = 9)	0.0 (0.0–1.2)	0.114	0.0 (0.0–1.2)	0.299	0.58 (0.10–1.71)	0.299	5 (2–7)	0.008^*∗*^
	PPR (*n* = 7)	0.4 (0.0–1.2)		0.4 (0.0–5.2)		0.89 (0.37–1.69)		7 (6–15)	
	PCR (*n* = 6)	0.0 (0.0–1.0)	0.118	0.0 (0.0–0.2)	0.181	0.55 (0.10–1.25)	0.220	5.5 (2–7)	0.181
	Non PCR (*n* = 10)	0.3 (0.0–1.2)		0.3 (0.0–5.2)		0.77 (0.37–1.71)		6.5 (4–15)	

^(1)^Blood: data previously published (Verma et al., 2013 [21]); ^(2)^LLABCs: large and locally advanced breast cancers; ^(3)^NAC: neoadjuvant chemotherapy; ^(4)^average cell count per 400x high-power field; ^(5)^AbN: absolute number (cells/mm^3^); ^(6)^Mann–Whitney *U* test; ^(7)^GPR (good pathological response, grades 5 and 4): no residual invasive disease, >90% loss of invasive disease, respectively; ^(8)^PPR (poor pathological response, grades 3, 2, and 1): 30–90% loss of invasive disease, <30% loss of invasive disease, and no loss of tumour cells, respectively; ^(9)^PCR (pathological complete response, grade 5): no residual invasive disease; ^*∗*^statistically significant.

**Table 8:** Expression of cytokines and PD-L1^(1)^ in LLABCs^(2)^ (pre-NAC and post-NAC^(3)^) and pathological complete response elicited in tumours following NAC.

Cytokines and PD-L1 (*n* = 16)		Pre-NAC				Post-NAC			
		Low/negative expression (*n*)	High expression (*n*)	Pearson chi-square value (PCR^(4)^ versus non-PCR)	*p* value	Low/negative expression (*n*)	High expression (*n*)	Pearson chi-square value (PCR versus non-PCR)	*p* value
IL-1	PCR (*n* = 6)	1	5	0.950	0.330	3	3	0.640	0.424
	Non PCR (*n* = 10)	4	6			3	7		

IL-2	PCR (*n* = 6)	2	4	0.019	0.889	3	3	1.571	0.210
	Non PCR (*n* = 10)	3	7			8	2		

IFN-*γ*	PCR (*n* = 6)	0	6	1.371	0.242	2	4	0.71	0.790
	Non PCR (*n* = 10)	2	8			4	6		

IL-4	PCR (*n* = 6)	1	5	0.152	0.696	4	2	0.423	0.515
	Non PCR (*n* = 10)	1	9			5	5		

IL-10	PCR (*n* = 6)	2	4	0.071	0.790	5	1	4.267	0.039^*∗*^
	Non PCR (*n* = 10)	4	6			3	7		

IL-17	PCR (*n* = 6)	2	4	0.019	0.889	5	1	6.112	0.013^*∗*^
	Non PCR (*n* = 10)	3	7			2	8		

TGF-*β* ^(5)^	PCR (*n* = 6)	4	2	0.423	0.515	4	2	3.484	0.062
	Non PCR (*n* = 10)	5	5			2	8		

PD-L1	PCR (*n* = 6)	3	3	0.640	0.424	4	2	0.019	0.889
	Non PCR (*n* = 10)	3	7			7	3		

^(1)^PD-L1: programmed death ligand 1; ^(2)^LLABCs: large and locally advanced breast cancers; ^(3)^NAC: neoadjuvant chemotherapy; ^(4)^PCR (pathological complete response, grade 5: no residual invasive disease); ^(5)^TGF-*β*: scored as negative or positive; ^*∗*^statistically significant.

**Table 9:** Clinical and pathological parameters of patients (*n* = 33) studied and the presence of pre-NAC^(1)^ tumour-infiltrating CD4^+^ and CD8^+^ and FOXP3^+^ T cells.

Groups	*N*	CD4^+^ T cells		CD8^+^ T cells		FOXP3^+^ T cells	
		Intratumoural Median (range)^(2)^ [*p* value^(3)^]	Stromal Median (range) [*p* value]	Intratumoural Median (range) [*p* value]	Stromal Median (range) [*p* value]	Intratumoural Median (range) [*p* value]	Stromal Median (range) [*p* value]
Age (years)							
<50	14	16.9 (1.4–166.2)	20.0 (1.0–162.2)	24.1 (0.8–97.4)	17.7 (1.8–110.0)	5.6 (0.4–26.2)	12.6 (0.8–26.8)
≥50	19	8.8 (0.6–171.0)	16.8 (1.0–242.0)	14.4 (0.4–202.4)	26.2 (2.0–201.6)	4.8 (0.8–96.8)	11.2 (0.8–110.6)
		[0.653]	[0.957]	[1.000]	[0.397]	[0.957]	[0.900]

BMI^(4)^ (kg/m^2^)							
≤30	20	7.6 (0.6–166.2)	16.4 (1.0–190.4)	20.3 (0.4–202.4)	22.8 (2.0–127.2)	6.3 (0.8–96.8)	14.3 (0.8–110.6)
>30	13	12.4 (1.4–171.0)	19.2 (1.0–242.0)	14.4 (0.8–197.2)	22.4 (1.8–201.6)	4.8 (0.4–60.4)	6.6 (0.8–27.8)
		[0.524]	[0.842]	[0.899]	[0.730]	[0.703]	[0.118]

Menopausal							
Pre	16	39.4 (1.4–171.0)	33.0 (1.0–242.0)	31.7 (0.8–197.2)	41.8 (1.8–201.6)	9.1 (0.4–60.4)	14.7 (0.8–27.8)
Post	17	6.4 (0.6–158.4)	15.8 (1.0–190.4)	12.8 (0.4–202.4)	22.4 (2.0–127.2)	4.6 (0.8–96.8)	6.6 (0.8–110.6)
		[0.191]	[0.204]	[0.157]	[0.958]	[0.326]	[0.423]

Tumour size							
<40 mm	18	7.6 (1.4–171.0)	17.3 (1.0–242.0)	20.8 (0.8–202.4)	22.8 (1.8–201.6)	4.4 (0.8–77.0)	9.3 (0.8–110.6)
≥40 mm	15	12.4 (0.6–129.0)	16.8 (1.0–162.2)	19.4 (0.4–99.2)	22.4 (3.4–114.0)	11.2 (0.4–96.8)	14.2 (0.8–44.8)
		[0.708]	[0.929]	[0.817]	[0.901]	[0.190]	[0.486]

Nodal status							
Negative	10	13.1 (3.6–171.0)	54.1 (9.0–242.0)	13.9 (3.4–202.4)	43.6 (1.8–201.6)	8.8 (2.4–77.0)	12.7 (3.0–110.6)
Positive	23	8.8 (0.6–166.2)	16.8 (1.0–162.2)	21.2 (0.4–112.6)	18.4 (2.0–118.8)	5.6 (0.4–96.8)	10.0 (0.8–32.0)
		[0.475]	[0.144]	[0.576]	[0.603]	[0.144]	[0.221]

Tumour grade							
1 (low)	2	47.6 (17.0–78.2)	106.5 (104–109)	64.0 (28.8–99.2)	88.0 (87.2–88.8)	32.5 (19.4–45.6)	35.8 (26.8–44.8)
2 (moderate)	13	4.8 (1.4–166.2)	10.4 (1.0–113.0)	10.4 (0.8–78.4)	11.2 (2.6–110.0)	4.2 (0.4–26.2)	5.2 (0.8–21.8)
3 (high)	18	22.7 (0.6–171.0)	37.5 (2.6–242.0)	31.9 (0.4–202.4)	70.5 (1.8–199.8)	6.3 (0.8–96.8)	14.0 (0.8–110.6)
		[0.026^(5)*∗*^]	[0.004^*∗*^]	[0.038^*∗*^]	[0.032^*∗*^]	[0.109]	[0.018^*∗*^]

ER^(6)^ status							
Negative	11	16.8 (5.8–158.4)	43.0 (5.2–190.4)	29.6 (1.0–202.4)	65.2 (2.0–127.2)	5.6 (0.8–96.8)	11.2 (0.8–110.6)
Positive	22	5.8 (0.6–171.0)	14.5 (1.0–242.0)	13.7 (0.4–197.2)	18.9 (1.8–201.6)	5.5 (0.4–60.4)	11.2 (0.8–44.8)
		[0.105]	[0.089]	[0.281]	[0.440]	[0.721]	[0.866]

HER-2 status							
Negative	23	12.4 (0.6–171.0)	16.0 (1.0–242.0)	19.4 (0.4–202.4)	26.2 (2.6–201.6)	7.4 (0.4–96.8)	11.6 (0.8–110.6)
Positive	10	7.6 (3.6–66.8)	20.0 (5.2–119.2)	14.3 (1.0–97.4)	15.0 (1.8–86.4)	3.6 (0.8–11.6)	9.3 (0.8–17.4)
		[0.658]	[0.658]	[0.428]	[0.133]	[0.114]	[0.221]

NAC regimen							
AC-TX^(7)^	16	12.8 (1.4–171.0)	20.3 (1.0–242.0)	25.4 (0.8–197.2)	47.8 (2.6–201.6)	7.2 (0.4–60.4)	12.7 (0.8–44.8)
AC-T	17	8.8 (0.6–166.2)	15.8 (2.6–190.4)	13.4 (0.4–202.4)	19.4 (1.8–127.2)	4.8 (0.8–96.8)	10.8 (0.8–110.6)
		[0.873]	[0.901]	[0.326]	[0.657]	[0.929]	[0.817]

^(1)^NAC: neoadjuvant chemotherapy; ^(2)^average cell count per 400x high-power field; ^(3)^Mann–Whitney *U* test; ^(4)^BMI: body mass index (≤30: nonobese, >30: obese);  ^(5)^Kruskal-Wallis test; ^(6)^ER: oestrogen receptor; ^(7)^AC-TX: doxorubicin, cyclophosphamide, taxotere, and Xeloda® (capecitabine), respectively; ^*∗*^statistically significant.

**Table 10:** Univariate and multivariate (logistic regression) analyses of clinicopathological parameters as predictive factors for pathological complete response to NAC^(1)^ in LLABCs^(2)^ (*n* = 33).

Parameters	Univariate analysis			Multivariate analysis		
	OR^(3)^	95% CI^(4)^	*p* value	OR	95% CI	*p* value
TILs^(5)^: high (LPBC^(6)^) versus low	20.22	3.45–118.65	0.001^*∗*^	11.17	1.41–88.49	0.022^*∗*^
Age: <50 versus ≥50	0.68	0.17–2.71	0.579	NA	NA	NA
Tumour size: <40 mm versus ≥40 mm	1.14	0.29–4.51	0.849	NA	NA	NA
Tumour grade: 3 versus 1/2	10.4	2.03–53.20	0.005^*∗*^	2.99	0.33–27.00	0.328
ER^(7)^ status: negative versus positive	4.67	0.96–22.79	0.049^*∗*^	1.01	0.11–9.63	0.994
HER-2 status: positive versus negative	1.95	0.43–8.83	0.386	NA	NA	NA
NAC regimen: AC-TX^(8)^ versus AC-T	3.06	0.74–12.63	0.123	NA	NA	NA

^(1)^NAC: neoadjuvant chemotherapy; ^(2)^LLABCs: large and locally advanced breast cancers; ^(3)^OR: odds ratio; ^(4)^CI: confidence interval; ^(5)^TILs: tumour-infiltrating lymphocytes; ^(6)^LPBC: lymphocyte-predominant breast cancer; ^(7)^ER: oestrogen receptor; ^(8)^AC-TX: doxorubicin, cyclophosphamide, taxotere, and Xeloda (capecitabine), respectively; ^*∗*^statistically significant; NA: not applicable.

## Abbreviations

5-FU: 5-Fluorouracil
A: Adriamycin
AbN: Absolute number
ALN: Axillary lymph node
C: Cyclophosphamide
CD: Cluster of differentiation
CTL: Cytotoxic T lymphocyte
CTLA-4: Cytotoxic T lymphocyte antigen 4
DAB: Diaminobenzidine
DFS: Disease-free survival
DC: Dendritic cell
DCIS: Ductal carcinoma* in situ*
ER: Oestrogen receptor
FOXP3: Forkhead box protein 3
HER2: Human epidermal growth factor receptor 2
HMGB1: High-mobility group box 1
HPF: High-power field
HRP: Horseradish peroxidase
H&E: Haematoxylin and eosin
IHC: Immunohistochemistry
IL: Interleukin
IFN-*γ*: Interferon-gamma
Itu-Ly: Intratumoural lymphocyte
LLABC: Large locally advanced breast cancer
MAb: Monoclonal antibody
MHC: Major histocompatibility complex
NAC: Neoadjuvant chemotherapy
NK: Natural killer
OS: Overall survival
pCR: Pathological complete response
PD-1: Programmed death 1
PD-L1: Programmed death ligand 1
RT: Room temperature
Str-Ly: Stromal lymphocyte
T: Docetaxel
TAA: Tumour-associated antigen
Th: T helper
Treg: T regulatory cell
TCR: T cell receptor
TGF-*β*: Transforming growth factor-beta
TIL: Tumour-infiltrating lymphocyte
X: Capecitabine.
