# Supplementary material for: Crucial Contributions by T Lymphocytes (Effector, Regulatory, and Checkpoint Inhibitor) and Cytokines (TH1, TH2, and TH17) to a Pathological Complete Response Induced by Neoadjuvant Chemotherapy in Women with Breast Cancer
Source: J Immunol Res. 2016 Sep 29;2016:4757405. doi: 10.1155/2016/4757405 (PMC5061970; doi:10.1155/2016/4757405)
Supplement: Supplementary file 1 — Additional file 1: documents the impact of NAC on the levels of TILs between pre- and post-NAC samples. The levels of both intra-tumoural and stromal TILs were not significantly altered, when pre-NAC samples were compared with post-NAC samples. Five out of 16 patients with a high level of TILs subsequently had a low level after NAC, whilst 1 out of 16 with a low level of TILs had a higher level post-NAC (p = 0.219). Additional file 2: prior to NAC, there was no significant correlation between the levels of circulating Tregs and those in the tumour microenvironment. Following NAC, however, there was a significant positive correlation between the % of circulating and intra-tumoural FOXP3⁺ Tregs. [Correlation Coefficient (rho) 0.687, p = 0.003]. Additional file 3: shows no significant correlation between circulating and tumour-infiltrating CTLA-4⁺ Tregs. Additional file 4: similar to pre-NAC tumour-infiltrating Tregs (FOXP3⁺ and CTLA-4⁺), the levels of pre-NAC circulating Tregs (AbNs and %) were not significantly different in any of the NAC response groups (GPR versus PPR and pCR versus non pCR, p > 0.05). Additional file 5: illustrates the effect of NAC on the expression of cytokines and PD-L1 in breast cancers. There was no significant difference between pre- and post-NAC expression (p > 0.05) except for IL-4. The expression of IL-4 following NAC was significantly reduced (p = 0.016); in 43.8% (7 out of 16) from high to low and in no case was this reversed. [file 4757405.f1.docx]

| **Additional file 1: Table A1 Alteration of the Levels of Tumour-infiltrating Lymphocytes (TILs) in LLABCs^(1)^ Undergoing NAC^(2)^** | | | | | |
| --- | --- | --- | --- | --- | --- |
| **Groups** | | | **Post NAC** | | **P Value^(3)^ (Pre- versus Post-NAC)** |
|  |  |  | **Low Infiltraion (n)** | **High Infiltration (n)** |  |
| TILs: Intra-tumoural (n=16) | Pre-NAC | Low Infiltraion (n) | 10 | 1 | 0.219 |
|  |  | High Infiltration (n) | 5 | 0 |  |
| TILs: Stromal (n=16) |  |  |  |  |  |
|  | Pre-NAC | Low Infiltraion (n) | 9 | 1 | 0.219 |
|  |  | High Infiltration (n) | 5 | 1 |  |
|  |  |  |  |  |  |
| ^(1)^ LLABCs: Large and locally advanced breast cancers; ^(2)^ NAC: Neoadjuvant chemotherapy; ^(3)^ Related-Samples McNemar Test | | | | | |

The impact of NAC on the levels of TILs is demonstrated by the changes in the level of TILs between pre- and post-NAC samples. The levels of both intra-tumoural TILs and stromal TILs were not significantly altered, when pre-NAC samples were compared with post-NAC samples. Five out of 16 patients with a high level of TILs were subsequently altered to a low level of TILs after NAC whilst 1 out of 16 changed from a low to a high level of TILs (p=0.219). This finding shows that NAC did not significantly affect the total TILs, albeit the number with high levels of infiltration was less in the post-NAC samples.

| **Additional File 2: Table A2 Correlation Between Circulating and Tumour-infiltrating FOXP3^+^ Tregs [Spearman's Correlation Coefficient (rho)] in Patients with LLABCs^(1)^ Undergoing NAC^(2)^ (n=16)** | | | | | | |
| --- | --- | --- | --- | --- | --- | --- |
| **Groups** | | **Pre-NAC Breast** | |  | **Post-NAC Breast** | |
|  |  | **Intra-tumoural infiltrating** | **Stromal infiltrating** |  | **Intra-tumoural infiltrating** | **Stromal infiltrating** |
| **Pre-NAC** | **%Circulating** |  |  |  |  |  |
|  | Correlation Coefficient | -0.116 | -0.208 |  | 0.024 | -0.064 |
|  | P Value (2-tailed) | 0.668 | 0.440 |  | 0.952 | 0.815 |
|  | **AbN Circulating** |  |  |  |  |  |
|  | Correlation Coefficient | -0.191 | -0.263 |  | -0.470 | -0.263 |
|  | P Value (2-tailed) | 0.478 | 0.325 |  | 0.066 | 0.325 |
| **Post-NAC** | **%Circulating** |  |  |  |  |  |
|  | Correlation Coefficient | 0.139 | 0.250 |  | 0.687 | 0.347 |
|  | P Value (2-tailed) | 0.607 | 0.350 |  | 0.003* | 0.188 |
|  | **AbN Circulating** |  |  |  |  |  |
|  | Correlation Coefficient | -0.022 | 0.068 |  | -0.342 | 0.016 |
|  | P Value (2-tailed) | 0.935 | 0.803 |  | 0.195 | 0.952 |
|  |  |  |  |  |  |  |
| ^(1)^ LLABCs: Large and locally advanced breast cancers; ^(2)^ NAC: Neoadjuvant chemotherapy; * Statistically significant | | | | | | |

Prior to NAC, the level of circulating Tregs was not found to be significantly correlated with the level of Tregs in the tumour microenvironment. However, this correlation became significant after NAC. There was a positive correlation between post-NAC % of peripheral circulating FOXP3⁺ Tregs and post-NAC intra-tumoural FOXP3⁺ Tregs [Correlation Coefficient (rho) 0.687, p=0.003]. Patients with a higher % of peripheral circulating FOXP3⁺ Tregs after NAC had high FOXP3⁺ Tregs in the residual tumours.

| **Additional File 3: Table A3 Correlation Between Circulating and Tumour-infiltrating CTLA-4^+^ Tregs [Spearman's Correlation Coefficient (rho)] in Patients with LLABCs^(1)^ Undergoing NAC^(2)^ (n=16)** | | | | | | |
| --- | --- | --- | --- | --- | --- | --- |
| **Groups** | | **Pre-NAC Breast** | |  | **Post-NAC Breast** | |
|  |  | **Intra-tumoural infiltrating** | **Stromal infiltrating** |  | **Intra-tumoural infiltrating** | **Stromal infiltrating** |
| **Pre-NAC** | **%Circulating** |  |  |  |  |  |
|  | Correlation Coefficient | -0.425 | -0.220 |  | 0.090 | 0.435 |
|  | P Value (2-tailed) | 0.101 | 0.412 |  | 0.741 | 0.092 |
|  | **AbN Circulating** |  |  |  |  |  |
|  | Correlation Coefficient | 0.055 | 0.242 |  | 0.473 | 0.317 |
|  | P Value (2-tailed) | 0.839 | 0.367 |  | 0.065 | 0.232 |
| **Post-NAC** | **%Circulating** |  |  |  |  |  |
|  | Correlation Coefficient | -0.405 | -0.146 |  | 0.145 | 0.305 |
|  | P Value (2-tailed) | 0.119 | 0.590 |  | 0.592 | 0.250 |
|  | **AbN Circulating** |  |  |  |  |  |
|  | Correlation Coefficient | -0.110 | -0.180 |  | 0.270 | 0.034 |
|  | P Value (2-tailed) | 0.685 | 0.506 |  | 0.312 | 0.899 |
|  |  |  |  |  |  |  |
| ^(1)^ LLABCs: Large and locally advanced breast cancers; ^(2)^ NAC: Neoadjuvant chemotherapy | | | | | | |

There was no significant correlation observed between circulating and tumour-infiltrating CTLA-4⁺ Tregs.

| **Additional File 4: Table A4 Blood^(1)^ and Tumour-infiltrating FOXP3^+^ and CTLA-4^+^ Tregs (Pre-NAC) in Women with LLABCs^(2)^ and Pathological Response Elicited in Tumours Following NAC^(3)^** | | | | | | | | | |
| --- | --- | --- | --- | --- | --- | --- | --- | --- | --- |
| **Tregs** | **Groups** | **Intra-tumoural Median (Range)^(4)^** | **P Value^(3)^** | **Stromal Median (Range)** | **P Value^(3)^** | **% Circulating Median (Range)** | **P Value^(3)^** | **AbN Circulating Median (Range)^(5)^** | **P Value^(6)^** |
| **FOXP3^+^** | GPR (n=9)^(7)^ | 12.8 (2.4-96.8) | 0.606 | 13.8 (2.2-110.6) | 0.606 | 1.50 (0.62-3.40) | 0.536 | 235 (107-427) | 0.071 |
|  | PPR (n=7)^(8)^ | 16.8 (4.2-45.6) |  | 17.4 (6.6-44.8) |  | 2.17 (1.18-3.24) |  | 165 (155-180) |  |
|  | PCR (n=6)^(9)^ | 36.6 (2.4-96.8) | 0.492 | 18.0 (5.2-110.6) | 0.562 | 1.55 (1.10-3.24) | 0.958 | 266 (107-427) | 0.181 |
|  | Non PCR (n=10) | 14.1 (4.2-45.6) |  | 15.9 (2.2-44.8) |  | 1.85 (0.62-3.40) |  | 168 (155-235) |  |
| **CTLA-4^+^** | GPR (n=9) | 0.4 (0.0-4.0) | 0.470 | 0.6 (0.2-10.0) | 0.606 | 1.10 (0.05-3.24) | 0.837 | 13 (5-19) | 0.174 |
|  | PPR (n=7) | 0.4 (0.0-2.2) |  | 0.6 (0.2-1.6) |  | 1.35 (0.76-1.71) |  | 17 (8.5-19) |  |
|  | PCR (n=6) | 1.1 (0.2-4.0) | 0.147 | 1.4 (0.2-10.0) | 0.093 | 1.05 (0.05-3.24) | 0.635 | 15 (6-19) | 0.875 |
|  | Non PCR (n=10) | 0.3 (0.0-2.2) |  | 0.4 (0.2-1.6) |  | 1.46 (0.23-1.80) |  | 14.5 (5-19) |  |
|  |  |  |  |  |  |  |  |  |  |
| ^(1)^ Blood: Data previously published (Verma et al, 2013); ^(2)^ LLABCs: Large and locally advanced breast cancers; ^(3)^ NAC: Neoadjuvant chemotherapy; ^(4)^ Average cell count per 400x high-power field; ^(5)^ AbN: Absolute number (cells/mm^3^); ^(6)^ Mann-Whitney U test; ^(7)^ GPR (good pathological response, grade 5 and 4): No residual invasive disease, >90% loss of invasive disease, respectively; ^(8)^ PPR (poor pathological response, grade 3, 2 and 1): 30-90% loss of invasive disease, <30% loss of invasive disease and no loss of tumour cells, respectively; ^(9)^ PCR (pathological complete response, grade 5): No residual invasive disease | | | | | | | | | |

Similar to pre-NAC tumour-infiltrating Tregs (FOXP3⁺ and CTLA-4⁺), the levels of pre-NAC circulating Tregs [absolute numbers (AbNs) and %] were not significantly different in any of the different NAC response groups (GPR versus PPR and pCR versus non pCR, p>0.05).

| **Additional File 5: Table A5 Expression of Cytokines and PD-L1^(1)^ in LLABCs^(2)^ Undergoing NAC^(3)^** | | | | | |
| --- | --- | --- | --- | --- | --- |
| **Cytokines** | | | **Post-NAC** | | **P Value^(4)^** |
|  |  |  | **Low/Negative Expression (n)** | **High Expression (n)** |  |
| IL-1 | Pre-NAC | Low/Negative Expression (n) | 3 | 2 | 1.000 |
|  |  | High Expression (n) | 3 | 8 |  |
| IL-2 | Pre-NAC | Low/Negative Expression (n) | 4 | 1 | 0.070 |
|  |  | High Expression (n) | 7 | 4 |  |
| IFN-γ | Pre-NAC | Low/Negative Expression (n) | 0 | 2 | 0.289 |
|  |  | High Expression (n) | 6 | 8 |  |
| IL-4 | Pre-NAC | Low/Negative Expression (n) | 2 | 0 | 0.016* |
|  |  | High Expression (n) | 7 | 7 |  |
| IL-10 | Pre-NAC | Low/Negative Expression (n) | 3 | 3 | 0.727 |
|  |  | High Expression (n) | 5 | 5 |  |
| IL-17 | Pre-NAC | Low/Negative Expression (n) | 3 | 2 | 0.688 |
|  |  | High Expression (n) | 4 | 7 |  |
| TGF-β^(5)^ | Pre-NAC | Low/Negative Expression (n) | 4 | 5 | 0.453 |
|  |  | High Expression (n) | 2 | 5 |  |
| PD-L1 | Pre-NAC | Low/Negative Expression (n) | 5 | 1 | 0.125 |
|  |  | High Expression (n) | 6 | 4 |  |
| ^(1)^ PD-L1: Programmed death ligand 1; ^(2)^ LLABCs: Large and locally advanced breast cancers; ^(3)^ NAC: Neoadjuvant chemotherapy; ^(4)^ Related-Samples McNemar Test; ^(5)^ TGF-β: Scored as negative or positive; * Statistically significant | | | | | |

Table A5 illustrates the effect of NAC on the alteration of the expression of cytokines and PD-L1 in breast cancers. There was no significant different observed between levels of expression pre- and post-NAC (p>0.05) except IL-4. The expression of IL-4 following NAC was significantly altered (p=0.016). In 43.8% (7 out of 16) of cases, the level of expression was altered from high (pre-NAC) to low/negative (post-NAC). In none of the cases (0 out of 16) was the level of expression altered from low/negative (pre-NAC) to high (post-NAC). Thus, the level of IL-4 expression in breast tumours was significantly reduced with NAC.
